# Stereoelectronic Effect of Protecting Groups on the Stability of Galactosyl Donor Intermediates

**DOI:** 10.3390/molecules30020218

**Published:** 2025-01-07

**Authors:** Ryan W. Kwok, Ryan Rutkoski, Pavel Nagorny, Mateusz Marianski

**Affiliations:** 1 Department of Chemistry, Hunter College, The City University of New York, 695 Park Ave., New York, NY 10065, USA; rkwok@gradcenter.cuny.edu; 2 PhD Program in Chemistry, The Graduate Center, The City University of New York, 365th Ave., New York, NY 10016, USA; 3 Department of Chemistry, University of Michigan, 930 University Ave., Ann Arbor, MI 48109, USA; rutkoski@med.umich.edu

**Keywords:** glycosylation mechanism, DFT, remote participation

## Abstract

Using methods of DFT, we investigated the effect of electron withdrawing and electron donating groups on the relative stability of tentative glycosyl donor reaction intermediates. The calculation shows that by changing the stereoelectronic properties of the protecting group, we can influence the stability of the dioxolenium type of intermediates by up to 10 kcal mol^−1^, and that by increasing nucleophillicity of the 4-*O*-Bz group, the dioxolenium intermediate becomes more stable than a triflate–donor pair. We exploited this mechanism to design galactosyl donors with custom protecting groups on O2 and O4, and investigated the outcome of the reaction with cyclohexanol. The reaction showed no change in the product distribution, which suggests that the neighboring group participation takes precedence over remote group participation due to kinetic barriers.

## 1. Introduction

Carbohydrates—or glycans—are responsible for communicating complex information about the state of a cell to its biological milieu [1]. By mimicking the functions of natural oligosaccharides, carbohydrate-based molecules have given rise to over 170 commercial drugs and vaccines [2]. Such applications, however, remain niche on account of their complex and time-consuming synthesis, which necessitates scrupulous regio- and stereocontrol of glycosidic bond formations. Improving existing strategies to facilitate the formation of stereo-specific glycosidic linkages would open new avenues in therapeutics, chemical sensing, and materials science [2,3].

A glycosylation reaction couples a glycosyl donor and acceptor, and proceeds through an idiosyncratic glycosyl donor intermediate. Rather than adopting a single well-defined structure, this intermediate—together with its counter ion—exists as an ensemble of structures that range from a covalently bound ion pair to a solvent-separated ion pair, and the oxocarbenium ion [4,5]. Understanding how to influence this ensemble and direct the reaction pathway towards a desired product is the main objective of mechanistic studies of the glycosylation reaction. In principle, the stereochemical control over the formation of a new glycosidic linkage can be imposed through the use of protecting groups on a glycosyl donor or acceptor, adjusting the nucleophillicity of the acceptor, properties of the leaving group, counter-ion, activator, or modifications to the reaction context via solvent and temperature [6,7]. Unfortunately, the fleeting nature of the reaction intermediates makes their direct characterization only possible in strongly stabilizing environments, such as in superacids, as gas-phase ions, or as chemical derivatives [8,9,10,11,12]. These conditions however, bring the donors out of the context of a chemical reaction and limit opportunities for a systematic study of the mechanism. In effect, the impact of the factors determining the favored reaction mechanism is still poorly understood, and the optimization of the glycosylation reaction remains a largely empirical endeavor.

The stereoselective formation of trans-glycosidic linkages is most reliably achieved using neighboring group participation, which uses an acyl protecting group on O2, to orchestrate the direction of a nucleophillic attack on the anomeric carbon [10,13]. The reaction proceeds through an $S_{N}$1-like mechanism in which the ester group shields the cis-side of the anomeric carbon, favoring the formation of a trans glycosidic linkage with respect to the substituents on C1 and C2. The cis–glycosidic linkages constitute a more formidable challenge, and numerous strategies to install them, involving chiral auxiliaries, intramolecular rearrangements, or specific activators, have been devised [14,15,16,17,18]. Among these frequently time-consuming and multistep approaches, the strategy of remote protecting group participation, which uses a distal participating group to direct the selective formation of a cis-linkage, has received particular attention due to its relative simplicity [6].

Previously, we used a combination of Cryo-IR spectroscopy and DFT to demonstrate that the acetyl group at O2 causes the glycosyl donors to adopt a dioxolenium-type structure by forming a covalent bond with anomeric carbon [10]. In the following study, we showed that galactosyl donors, which carry the participating group at the remote C4 position, also form dioxolenium-type ions, which have the β-side of the anomeric carbon shielded by the covalently bound carbonyl group. Thus, the fingerprint of the dioxolenium-type ion in the IR spectrum was correlated with increased α-selectivity when coupling with 2-propanol [19]. When the acetyl group was shifted to O6, we observed a significant decrease in the reaction’s selectivity, which was assigned to the lack of shielding of the anomeric carbon and agreed with the lack of the dioxolenium-type signature in the IR spectrum. Other groups observed similar trends involving these protected glycosyl donor intermediates [11,20]. Recently, Pagel et al. also demonstrated, using same technique, that increasing the electron density of the participating group on C4 of a glycosyl donor correlates with the formation of the dioxolenium-type intermediates, thereby enhancing the α-selectivity of the donor [21].

Nevertheless, the exact mechanism of how distal protecting groups engage with the reaction mechanism is still debated within the scientific community [4,22,23]. It has been argued that the the formation of dioxolenium ions is kinetically unfavorable under the reaction conditions due to several high-energy steps required for the glycosyl donor to adopt this structure and that the dioxolenium-type ions are observed during the gas phase due to long equilibration times, which follows the thermal activation of the ions [12,22,24]. Instead, the stereoselectivity of the glycosyl donors arises from the electron-withdrawing properties of the protecting groups, which draw electron density away from the carbohydrate ring. In doing so, the distal protecting group destabilizes the oxocarbenium-type intermediate that would favor the non-selective $S_{N}$1-like mechanisms and shifts the reaction mechanism towards stereoselective $S_{N}$2-like mechanisms [22,25].

**Figure 1:**
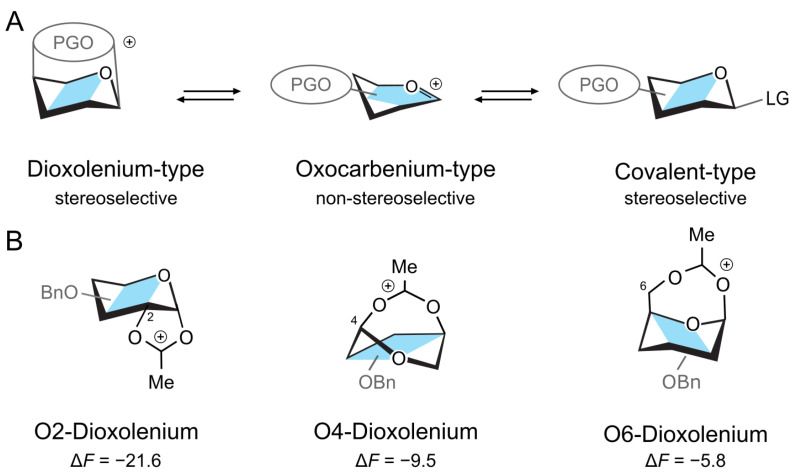
(**A**) Structures of a donor intermediate with a participating group (PG). The stereoelectronic properties of PG can shift the equilibrium between the intermediates. (**B**) The three dioxolenium-type ions and their relative stability (in kcal mol^−1^) in a gas phase with respect to the oxocarbenium structure.

Herein, we hypothesize that the glycosylation mechanism might depend on the electronic properties of the protecting groups on the glycosyl donor and that by modifying their electron withdrawing properties, it should be possible to differentiate between the two stereoselective mechanisms, the $S_{N}$2-dominant (ion-pair) and the $S_{N}$1-dominant (dioxolenium ion) reaction pathways (Figure 1A). The $S_{N}$1 pathway can be stabilized by increasing the nucleophillicity of the participating group through the addition of an electron donating group (EDG) to the benzoyl protecting group, whereas the $S_{N}$2 mechanism should be promoted by adding an electron withdrawing group (EWG). To demonstrate this principle, we employed density functional theory (DFT) calculations to systematically probe the effect of the position and strength of electron-withdrawing protecting groups on a glycosyl donor. DFT has been repeatedly tested and proven to be a viable means of mechanistic study, and has long been used to refine—or redefine—the mechanisms of organic reactions, such as $S_{N}$Ar substitutions [26,27], [2 + 2] photoorganic cycloadditions [28], and Diels–Alder reactions [29,30]. DFT has been also applied to investigate the conformational and structural stability of carbohydrates [24,31,32], determine glycosylation reaction mechanisms [5,19,33,34], study the interiors of enzymatic centers [35], or to validate specific glycosylation mechanisms [25,36,37,38,39,40,41,42,43,44], although only a limited number of DFT studies have attempted a systematic study of the factors that would influence reaction outcomes [33,45,46,47]. Understanding and capitalizing on protecting group-assistance would greatly alleviate the difficulty in predictive carbohydrate synthesis by allowing for the optimization of glycosyl donor building blocks [48,49,50].

The DFT calculations confirmed a correlation between the stereoelectronic properties of the protecting group and the stability of the intermediates. In particular, all O2- and O4-protected donors with electron donating groups will favor the dioxolenium-type intermediate over the triflate-bound covalent species as the lowest-energy reaction intermediate in DCM. To probe these predictions, we designed and synthesized two galactose donors that have participating groups with opposite electronic properties at the O2 and O4 positions, and examined the products of their coupling with cyclohexanol. We observed that the configuration of the newly formed glycosyl linkage does not correlate with the predicted thermal stability of the reaction intermediates but is determined by the participation with the protecting group at the O2 position. This observation suggests that, rather than relative thermal stability, it is the kinetic barriers associated with the energy needed to reorganize the donor or displace the counterion that determine the stereochemistry of the new linkage.

## 2. Results

First, we will consider the relative stability of differently protected dioxolenium- and oxocarbenium-type galactosyl ions in the gas phase. Herein, we rotated the position of the acetyl protecting group between O2, O4, and O6, while keeping the other positions perbenzylated. The calculations were performed using the PBE0 + D3/6-311 + G(d,p) level of theory in Gaussian16 and included harmonic free-energy corrections at 300 K [51,52,53]. The relative free energies, ΔF, are calculated as the difference between the free energies of a pair of conformers and represent the relative stability of the two species. The complete numerical data are available in the Supplementary Materials (Tables S1–S4). The DFT calculations showed that the acetyl participating group at O2 stabilizes the dioxolenium-type ion by ΔF=−21.6 kcal mol^−1^ with respect to the oxocarbenium-type ion (Figure 1B). Similar stabilization of the dioxolenium ion is observed for O4 participation, albeit to a lesser degree (ΔF=−9.5 kcal mol^−1^). Shifting the acetyl group to O6 further decreases the energy gap between the dioxolenium-type and oxocarbenium-type intermediates to ΔF=−5.8 kcal mol^−1^. Thus, the calculations confirm that the relative stability of the dioxolenium and oxocarbenium ions depends on the position of the protecting group. This relation can be assigned to the strain caused by different ring sizes—the five-member ring formed by the 2-*O*-acetylated ions has the least strain, while the seven-member ring formed by the 6-*O*-acetylated ions has the most strain.

Next, we investigated how the modification of the stereoelectronic properties of the protecting groups affects the relative stability of the two ion types. The calculations used the most-stable conformers of dioxolenium and oxocarbenium ions derived from previous research [19]. These structures were obtained by first using an evolutionary algorithm to sample the conformational space of oxocarbenium- and dioxolenium-type ions. The relative energy of these sampled structures were then computed using many-body dispersion-corrected hybrid DFT, PBE0 + MBD, as implemented in FHI, with the aim of determining the most stable conformers. For additional details, we invite interested readers to consult the relevant paper [19]. Changing the 4-*O*-acetyl to a 4-*O*-benzoyl participating group causes the free energy difference between the dioxolenium- and oxocarbenium-type ions, ΔF, to increase by −3.9 kcal mol^−1^ from −9.5 to −13.4. Substitutions at positions O2 and O6 also increase the stability of the dioxolenium ion by −5.8 and −2.7 kcal mol^−1^, respectively.

**Figure 2:**
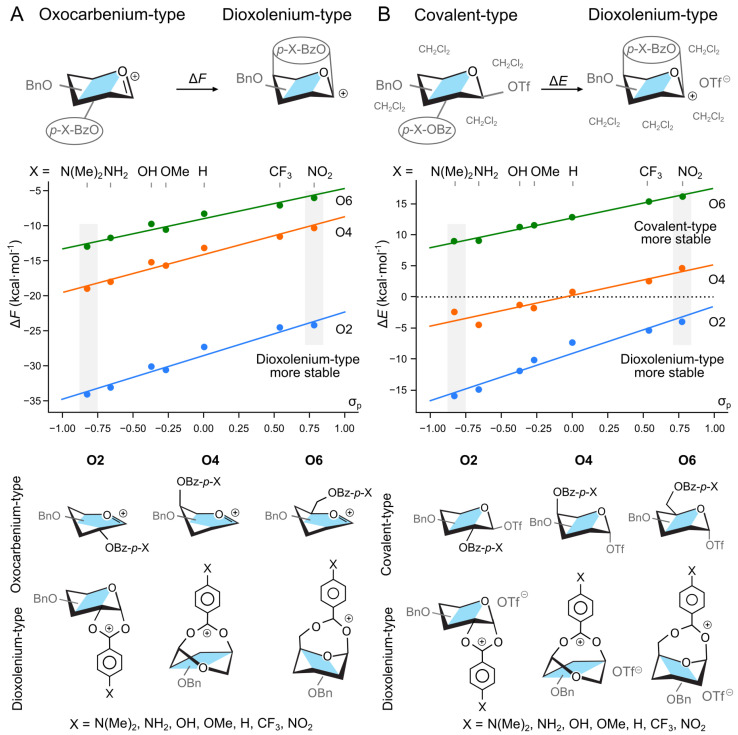
Relative stability of (**A**) oxocarbenium and dioxolenium intermediates in a gas phase, and (**B**) covalent triflate-donor and dioxolenium-type ion pair in hybrid PCM model as a function of the electron-withdrawing group on the participating benzoyl group. $\sigma_{p}$ indicates the substituent constant from the Hammett equation for para-substituted benzene. The energetics of structures highlighted with gray boxes are discussed in the text. The general structures of ions (gas phase) and ion-pairs (hybrid solvation) are shown below. Four explicit DCM molecules were omitted for clarity.

The energy difference between these ion types could be further fine-tuned through the attachment of chemical substituents to the aromatic ring. The calculations showed that the installation of electron donating substituents results in better stabilization of the dioxolenium-type ion, while the use of electron-withdrawing substituents destabilizes it (Figure 2A). For instance, the electron-withdrawing NO_2_ group at the para position of the 4-*O*-benzoyl group decreases the predicted ΔF between two species from −13.4 to −10.5 kcal mol^−1^. By contrast, the electron-rich N(Me)_2_ increases it to −19.2 kcal mol^−1^. Thus, by manipulating the electronic properties of the protecting group we can tune the relative stability of the O4 protected ions over the scale of 8.7 kcal mol^−1^. Similar trends are observed for O2 and O6 participation, where, for the same pair of substituents, the relative stability ranges from ΔF=−34.1 to −24.4 kcal mol^−1^, and ΔF=−13.2 to −6.1 kcal mol^−1^, respectively. This ability of protecting groups to stabilize the dioxolenium-type intermediate can be qualitatively estimated from the substituent constant, $\sigma_{p}$, used in the Hammett equation.

The effect of the EWG on the relative stability of the two ions also held when we expanded our system to include a counter ion and a hybrid solvent model. The triflate anion, OTf^−^, was selected as our counter ion due to its widespread use in the synthesis as a α-directing reagent. To account for the discrete binding of the glycosyl donor with solvent molecules, in addition to the polarizable continuous model (PCM) [54], we added four explicit molecules of CH_2_Cl_2_ in proximity to the triflate counter-ion (Figure 2B). These structures were optimized from multiple starting geometries and the most stable conformers were selected for analysis. Herein, we report relative energy, ΔE, due to large entropic penalty needed to localize explicit solvent molecules, and what is consistent with common practice [55]. Using a perbenzylated galactose donor with a benzoyl group at O2, O4, or O6, we reoptimized the covalently bound triflate intermediates, and two additional noncovalent intermediates—the dioxolenium type and contact–ion pair intermediates (Figure 2B). For these calculations, the triflate molecule was always placed on the side opposite of the anomeric carbon to the participating group. Under these conditions, the contact ion pair was not a stable minimum and always collapsed to the covalently bound species. We note, however, that triflate ions are indeed expected to form covalently bound intermediates, and DFT is able to yield contact–ion pairs for other counterions such as ${\mathrm{BF}}_{4}^{-}$ [56,57]. Next, using the optimized structures of triflate–donor pairs, we started to vary the para substituent of the participating benzoyl group.

In the case of donors with the O2-participating group, the solvated dioxolenium-type intermediates were consistently more stable than the covalently-bound species (Figure 2B), with the energetics ranging from ΔE=−4.0 kcal mol^−1^ for the NO_2_-substituted benzoyl group to ΔE=−16.0 kcal mol^−1^ for the N(Me)_2_-substituted. On the other hand, the solvated dioxolenium ions of the O6-benzoylated donors were always less stable than their covalently bound counterparts, with the dioxolenium ion of the most electron-donating group still being 9.0 kcal mol^−1^ less favorable. These trends are consistent with previously reported literature on the relative strengths of participation [20,21,22]. O4-benzoylated donors, however, displayed the most interesting behavior. For this position, the benzoyl protected dioxolenium ion is marginally less stable than the covalently bound triflate, but increasing the electron density of the protecting group by changing the substituents at the 4-*O*-benzoyl group can stabilize the dioxolenium-type intermediate by up to 4.5 kcal mol^−1^. Overall, the stability of the dioxolenium ion can be varied by 9.1 kcal mol^−1^ between the use of strong electron withdrawing and donating groups.

A similar trend holds for the transition state energies of the formation of the dioxolenium-type intermediate via a concerted nucleophillic attack of the carbonyl oxygen on the anomeric center and displacement of the leaving group (Table S2). The Cl^−^ leaving group was selected for the transition state calculations for two reasons. First, the halides are common leaving groups used in the glycosynthesis, and second, in comparison to OTf^−^, they drastically reduce the computational cost and complexity of the derivation of the transition state in the DFT calculations. Without the counter ion, the acetyl attaches to the C1 in a barrierless process. By including the chloride leaving group, the respective energy of the transition states for the displacement reactions are 28.6, 45.0, and 39.8 kcal mol^−1^ for O2-, O4-, and O6-acetylated donors respectively. The 16.4 kcal mol^−1^ difference between the transition states of O2- and O4-acetyl displacement reactions is most likely due to the unfavorable orientation of the carbonyl oxygen during the attack on the anomeric carbon. In the case of benzoyl-protected donors, the transition state energies can be further altered over a range of approximately 6 kcal mol^−1^ by changing the substituents (Table S2).

The predictions about the relative stability of the intermediates are in line with many observations in the literature about the effect of protecting group nucleophilicity on the reaction outcome. For instance, substituting the acetyl group with a benzoyl group results in increased α-selectivity of the benzoyl glycosyl donor, which correlates with the increased stability of the dioxolenium-type intermediate [19,20,21,58]. This effect has also been further explored by Greis et al., who reported, for glycosyl donors armed with electron-rich protecting groups, a correlation between the increased abundance of the dioxolenium-type ions observed with Cryo-IR spectroscopy and enhanced α-selectivity during glycosylation [21,59]. Conversely, Kim et al. observed that mannopyranosylations with the electron withdrawing o-trifluoromethylbenzenesulfonyl, benzylsulfonyl, p-nitrobenzoyl, benzoyl, or acetyl protecting groups at O3, O4, or O6 resulted in β-directing effects that similarly correlate with electron-withdrawing ability: here the former two groups possess higher selectivity than the others [60]. Furthermore, α-directing effects from electron-withdrawing groups have also been observed by Sun et al. They reported that the use of a 1-picolinyl-5-azido thiosialoside donor resulted in high α-selectivity during sialylation, which also has been attributed to the electron-withdrawing ability of picolinyl groups [61].

**Figure 3:**
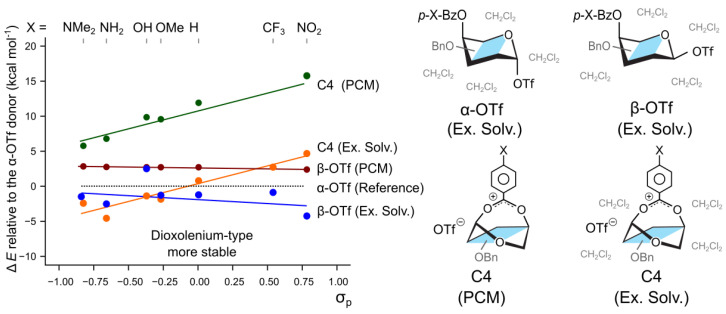
The relative stability of potential reactive intermediates with respect to covalently bound α-OTf in either PCM or explicit solvent (Ex. Solv.). The C4-participating dioxolenium ion becomes the most stable available species when methoxy substituent is added in the para position.

To compare the participation mechanism with the $S_{N}$2-like displacement of the leaving group, we calculated the effect of the substituents on the relative stability of the α- and β-protected triflate adducts of 4-*O*-Bz substituted galactosyl donors. It has been shown that the reaction follows the Curtin–Hammett regime and that the less stable β-triflates predominantly participate in the reaction, leading to the selective formation of the α-product [23]. The calculations using only the implicit PCM solvent model indeed agrees that the β-anomer of 4-*O*-Bz galactosyl donor is 2.7 kcal mol^−1^ less stable than the α-anomer (Figure 3). Changing the substituent on the para position of the protecting group from N(Me)_2_ to NO_2_ alters the energy difference between the α- and β-anomers by only 0.3 kcal mol^−1^, from 2.7 to 2.4 kcal mol^−1^ respectively. A similar trend is observed for the hybrid solvation model which also shows that the β-triflates are stabilized by the electron withdrawing groups. Therefore, the impact of the stereoelectronic groups on the equilibrium populations of the two triflate-bound donor anomers is a much smaller effect than the stabilization observed for the differently-protected dioxolenium ions (Figure 3). First, this trend appears to be opposite to that observed for the dioxolenium ions: here, the electron-withdrawing groups stabilize the β-anomers with respect to the α-anomer. Second, the C4-participating dioxolenium-type intermediates become the most stable species in a solution (Figure 3—C4, Ex. Solv.) for the *p*-MeO-Bz protecting group attached to O4. These observations confirm that the relative stability of dioxolenium-type and covalent intermediates can be modulated by the protecting groups.

**Figure 4:**
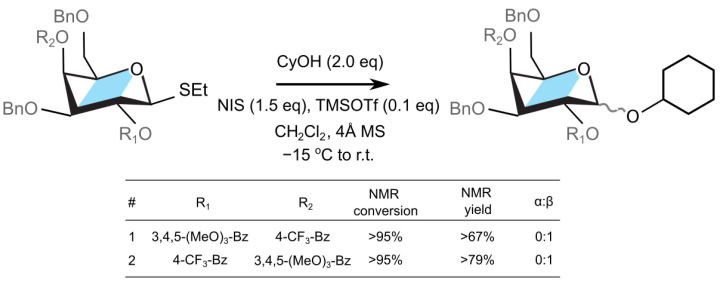
Coupling of a galactosyl donor designed with custom protecting groups R_1_ and R_2_.

Based on the calculations described so far, we hypothesized that the electron-donating substituent on the participating group could help stabilize the dioxolenium-type intermediate and promote the associated reaction pathway. To test this hypothesis, we designed a galactosyl donor with custom protecting groups on O2 and O4. We selected the galactosyl donor so that the protecting groups have opposite directing effects: the protecting group at O2 should direct the reaction towards a β-product via neighboring group participation, and the protecting group at O4 should shield the β-side and promote the formation of the α-product. By deactivating the O2-participating group with an EWG (*p*-CF_3_-Bz group) and activating the O4-participating group with an EDG (3,4,5-(MeO)_3_-Bz), we will alter the distribution of the two anomeric products resulting from coupling with cyclohexanol (Figure 4). The calculation performed on such custom glycosyl donors suggest that the O4-participating dioxolenium-type intermediate (ΔF=−15.7 kcal mol^−1^) is competitive with the O2-participation (ΔF=−15.4 kcal mol^−1^). Finally, we also inverted the two protecting groups to probe whether the participating group with an EDG substituent at the C2 position further affects β-selectivity.

The aforementioned galactose derivatives were synthesized (cf. Supporting Information) and employed as donors for the glycosylation reactions with cyclohexanol as an acceptor (cf. Figure 4). First, we explored the substrate containing an electron withdrawing benzoate (*p*-CF_3_-Bz) at the O4 position, and an electron rich benzoate group (3,4,5-(MeO)_3_-Bz) at the O2 position (Figure 4, entry **1**). Subjecting this donor and cyclohexanol (2 equiv) acceptor to the standard glycosylation conditions (NIH, TMSOTf) resulted in the complete consumption of the thioglycoside within 30 minutes and formation of the corresponding glycosylation product in 67% NMR yield (cf. Supplementary Materials). The NMR analysis of the crude reaction mixture revealed that the anomeric C1-proton has β-configuration ($J_{12}$ = 9.9 Hz) and that no α-anomer was observed. The observed stereolectivity is, as such, consistent with O2 participation by the 3,4,5-(MeO)_3_-Bz group. The electron donating methoxy substituents on benzoate stabilize the dioxolenium donor.

Following the experiment above, the donor containing deactivated *p*-CF_3_-Bz group on O2 and activated electron rich 3,4,5-(MeO)_3_-Bz group at the O4 position was investigated next (Figure 4, entry **2**). Subjecting this substrate to the reaction with cyclohexanol under the standard conditions (NIS, TMSOTf) led to the full consumption of the starting material within 30 minutes and formation of a single glycosylation product, as determined by the NMR analysis of the crude reaction mixture (79% NMR yield). The anomeric C1-proton of the observed glycosylation product was again assigned to be exclusively in the β-configuration ($J_{12}$ = 8.4 Hz). Thus, the configuration of the product suggested that the O2 *p*-CF_3_-Bz neighboring group participation remains the dominant reaction pathway, and that neither of the two α-directing mechanisms—the triflate–donor pair formation or the O4-participation—significantly contributed to the stereochemical outcome of this reaction. The former mechanism, the β-protected triflate–donor pair, could have been destabilized due to electrostatic interactions with the electron-rich protecting group at the O4 position [22], although the calculations predict only a mild decrease in the stability of 0.5 kcal mol^−1^ with respect to Bz group. In addition, the lack of the α-product due to the latter mechanism, via remote O4 group participation, can be attributed to either the large reaction barriers needed to reorganize the donor to form the dioxolenium complex, or that there is a lower transition state energy for the displacement of the leaving group from the donor by the neighboring protecting group [62,63]. This observation implies that, despite the difference in the thermodynamic stability, the neighboring group participation takes precedence before remote group participation due to either the lower kinetic barriers of the donor rearrangement or the displacement of the leaving group (Table S2).

## 3. Conclusions

In summary, we used DFT calculations to demonstrate the effect of stereoelectronic properties of a protecting group on the relative stability of the oxocarbenium, dioxolenium, and covalent-ion pairs. We observed that EDGs stabilizes the dioxolenium intermediate with respect to the oxocarbenium ion and triflate complex, whereas EWGs destabilize it. The effect, which ranges from 8 to 10 kcal mol^−1^ between N(Me)_2_ and NO_2_ groups is comparable for O2, O4, and O6 participation, and arises from the increased nucleophilicity of the carbonyl oxygen of the protecting group, which facilitates covalent bond formation with the anomeric carbon. Furthermore, the DFT calculations predict the following: (1) the EDG substituent on the O4-protecting group can render the dioxolenium-type intermediate in solution more stable than the covalently bound triflate ion pair; and (2) by adding EDGs to the C4-protecting group and EWGs to O2-protecting group, it should be possible to thermodynamically favor the O4-participation over O2.

Next, we tested these predictions by designing custom galactosyl donors with modified protecting groups on O2 and O4 to both weaken and strengthen the participation. The calculations predicted that for this pair of substituents, the C4-dioxolenium type of the intermediate substituted with electron donating groups should be more stable than the C2-dioxolenium intermediate with electron withdrawing group. However, despite the C2-protecting group being deactivated with an electron-withdrawing CF_3_ substituent, the reaction with cyclohexanol produced only the β-product. This suggests that neighboring group participation will take precedence over remote group participation despite the comparable thermal stability of their intermediates. This is most likely due to large energy barriers associated with the reorganization of the donor and the displacement of the leaving group during O4 participation.

## Data Availability

Experimental details and structures of investigated conformers are available in the Supplementary Materials.

## Supplementary Materials

The following supporting information can be downloaded at: https://www.mdpi.com/article/10.3390/molecules30020218/s1, Tables S1–S4: Energetics of intermediates and transition states discussed in the manuscript; and detailed synthesis and characterization of substrates and products showed in Figure 4 [64].
