# Supplementary material for: Stereoelectronic Effect of Protecting Groups on the Stability of Galactosyl Donor Intermediates
Source: Molecules. 2025 Jan 7;30(2):218. doi: 10.3390/molecules30020218 (PMC11767833; doi:10.3390/molecules30020218)
Supplement: Supplementary file 1 [file molecules-30-00218-s001.zip › molecules-3281381-supplementary.pdf]

# Supporting Information

## Stereoelectronic effects on the participation mechanism in glycosylation reactions

Ryan W. Kwok [1,2], Ryan Rutkoski[3], Pavel Nagorny[3,*], and Mateusz Marianski[1,2,*]

[1] Department of Chemistry, Hunter College, The City University of New York, 695 Park Ave, New York, NY 10065, USA;

[2] PhD Program in Chemistry, The Graduate Center, The City University of New York, 365 th Ave, New York, NY 10016, USA

[3] Department of Chemistry, University of Michigan, 930 University Ave., Ann Arbor, MI 48109

[*] Correspondence: nagorny@umich.edu; mmarians@hunter.cuny.edu

## Table of Contents

1. Computational Details…………………………………………………………...S2
2. General Methods……………………………………………………………………S4
3. Preparation of New Compounds……………………………...…………………..S4
4. Studies of C2/C4 directed glycosylation of thioglycosides………………………..S9
5. NMR Spectra………………………………………………………………………S12

## Computational Details

Density-Functional Theory (DFT) was applied to investigate the energetics of the glycosyl ion intermediates. The energetics of the perbenzylated galactosyl dioxolenium ions relative to the oxocarbenium ions was first calculated in the gas phase, and again using four explicitly rendered dichloromethane molecules. (Tables S1-3). The reaction barriers for dioxolenium ion formation were calculated for a permethylated system with a chlorine leaving group to alleviate computational costs (Table S4). All energetics of the reactants, transition states and products were calculated at the PBE1PBE/6-311+G(d,p) level of theory using Gaussian16, ultrafine grid settings, and the default convergence criteria.

**Table S1:** Gas phase relative free energies of perbenzylated galactosyl dioxolenium ions for C2, C4 and C6 participation relative to their corresponding oxocarbenium ions. Free energies in kcal mol$^{-1}$

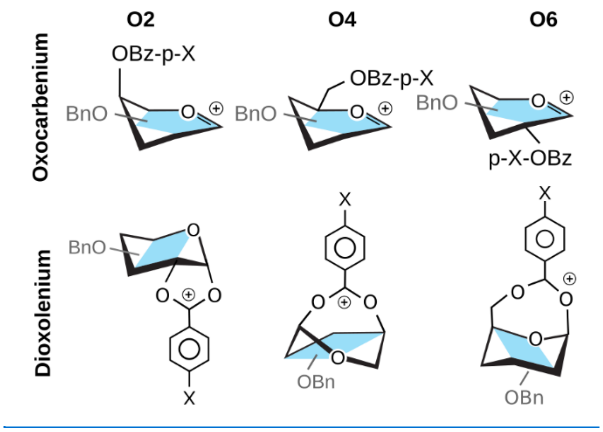


| R | σ | C2 | C4 | C6 |
|---|---|---|---|---|
| $N(Me)_2$ | -0.83 | -34.1 | -19.2 | -13.2 |
| $NH_2$ | -0.66 | -33.2 | -18.1 | -11.9 |
| OH | -0.37 | -30.3 | -15.4 | -10.0 |
| OMe | -0.27 | -30.7 | -15.9 | -10.6 |
| H | 0.0 | -27.4 | -13.4 | -8.5 |
| $CF_3$ | 0.54 | -24.8 | -11.6 | -7.3 |
| $NO_2$ | 0.78 | -24.4 | -10.5 | -6.1 |

**Table S2:** Relative free energy reaction barriers for per-methylated galactosyl dioxolenium ion formation with chlorine leaving groups. Free energies in kcal mol$^{-1}$

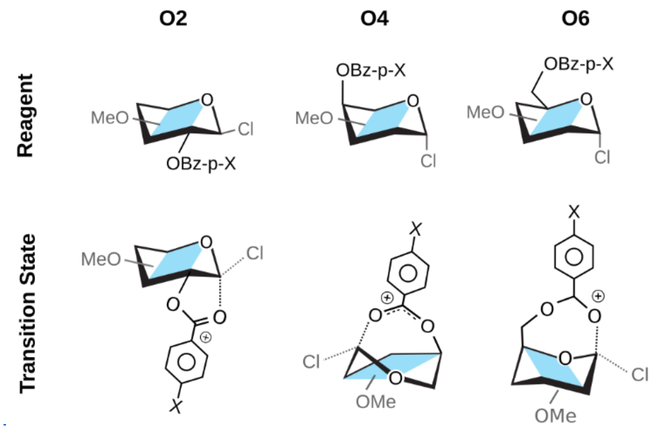


| R | σ | C2 | C4 | C6 |
|---|---|---|---|---|
| $N(Me)_2$ | -0.83 | 25.3 | 41.9 | 37.8 |
| $NH_2$ | -0.66 | 25.2 | 42.1 | 38.4 |
| OH | -0.37 | 28.5 | 43.8 | 39.5 |
| OMe | -0.27 | 28.0 | 43.4 | 39.1 |
| H | 0.0 | 28.6 | 45.0 | 39.8 |
| $CF_3$ | 0.54 | 30.5 | 46.8 | 41.1 |
| $NO_2$ | 0.78 | 31.3 | 47.8 | 42.6 |

**Table S3:** Explicit solvation relative energies of perbenzylated galactosyl dioxolenium ions for C2, C4 and C6 participation relative to their corresponding oxocarbenium ions. Energies in kcal $mol^{-1}$

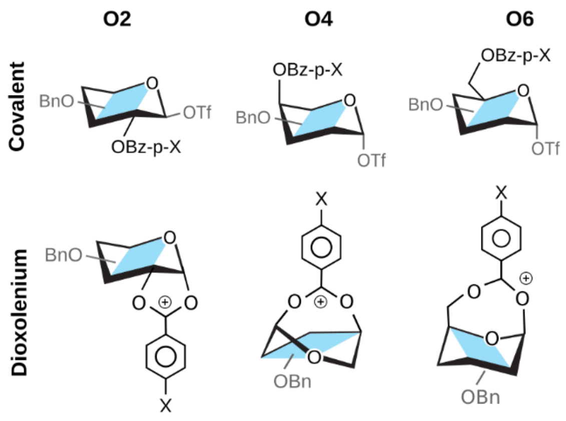


| R | σ | C2 | C4 | C6 |
|---|---|---|---|---|
| N(Me)2 | -0.83 | -16.0 | -2.4 | 8.9 |
| $NH_2$ | -0.66 | -15.0 | -4.5 | 9.0 |
| OH | -0.37 | -11.9 | -1.3 | 11.3 |
| OMe | -0.27 | -10.1 | -1.8 | 11.5 |
| H | 0.0 | -7.4 | 0.9 | 12.8 |
| $CF_3$ | 0.54 | -5.3 | 2.6 | 15.3 |
| $NO_2$ | 0.78 | -4.0 | 4.6 | 16.2 |

**Table S4:** Relative energies of the beta triflate contact ion-pair and the C4 dioxolenium ion with a triflate counterion relative to the alpha triflate contact ion-pair. The relative energetics of the dioxolenium ion for both the implicitly solvated and explicitly solvated systems are included. Energies in kcal $mol^{-1}$

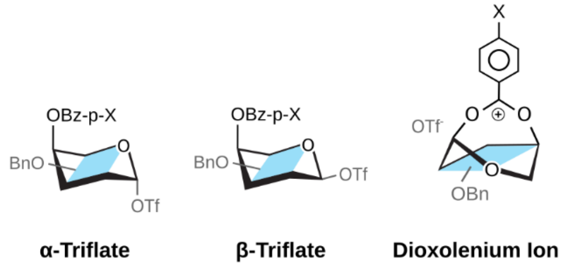

| R | σ | Implicitly Solvated Beta Triflate Contact-ion Pair | Implicitly Solvated Dioxolenium Ion | Explicitly Solvated Beta Triflate Contact-ion Pair | Explicitly Solvated Dioxolenium Ion |
|---|---|---|---|---|---|
| $N(Me)_2$ | -0.83 | 2.8 | 5.8 | -1.5 | -2.4 |
| $NH_2$ | -0.66 | 2.8 | 6.6 | -4.2 | -4.5 |
| OH | -0.37 | 2.7 | 9.8 | 2.6 | -1.3 |
| OMe | -0.27 | 2.7 | 9.5 | -1.3 | -1.8 |
| H | 0.0 | 2.7 | 11.9 | -1.2 | 0.9 |
| $CF_3$ | 0.54 | 2.6 | 14.5 | -0.9 | 2.6 |
| $NO_2$ | 0.78 | 2.4 | 15.7 | -4.1 | 4.6 |

**General Methods**

All reagents and solvents were purchased from commercial sources and were used as received without further purification unless otherwise specified. Ethyl-β-D-thiogalactopyranoside **(B.1)** was purchased from Combi-Blocks (Lot #B48996). $CH_2Cl_2$, $Et_2O$, DMF, PhMe. and THF were purified by Innovative Technology's Pure-Solve System using basic alumina. 4Å molecular sieves were activated prior to use by heating under reduced pressure. Cooling was achieved by use of Cryocool machine or ice/salt bath. Heating was achieved by use of a silicone oil bath with heating controlled by an electronic contact thermometer. Deionized water was used in the preparation of all aqueous solutions and for all aqueous extractions. Solvents used for extraction and chromatography were ACS or HPLC grade. Purification of reactions mixtures was performed by flash chromatography using SiliCycle SiliaFlash P60 (230-400 mesh). Yields indicate the isolated yield of the title compound with ≥95% purity as determined by $^1$H NMR analysis. Diastereomeric ratios were determined by $^1$H NMR analysis. Structural assignments were made with additional information from gHMBC experiments.

$^1$H NMR spectra were recorded on Varian vnmrs 700 (700 MHz), Varian vnmrs 500 (500 MHz), Varian MR400 (400 MHz), Bruker Avance Neo 500 (500 MHz) spectrometers and chemical shifts (δ) are reported in parts per million (ppm) with solvent resonance as the internal standard ($CDCl_3$ at δ 7.26, $C_6D_6$ at δ 7.16). Data are reported as (br = broad, s = singlet, d = doublet, t = triplet, q = quartet, m = multiplet; coupling constant(s) in Hz; integration). Proton-decoupled 13C NMR spectra were recorded on Varian vnmrs 700 (700 MHz), Varian vnmrs 500 (500 MHz), or Varian MR400 (400 MHz) spectrometers and chemical shifts (δ) are reported in ppm with solvent resonance as the internal standard ($CDCl_3$ at δ 77.16, $C_6D_6$ at 128.06). High resolution mass spectra (HRMS) were recorded on Agilent 6230 TOF or VG (Micromass) 70-250-S Magnetic sector mass spectrometers in the University of Michigan mass

spectrometry laboratory. Optical rotations were measured in a solvent of choice on a JASCO P-2000 polarimeter at 589 nm (D-line).

**Summary of the Synthesis of Substrates in Figure 4.**

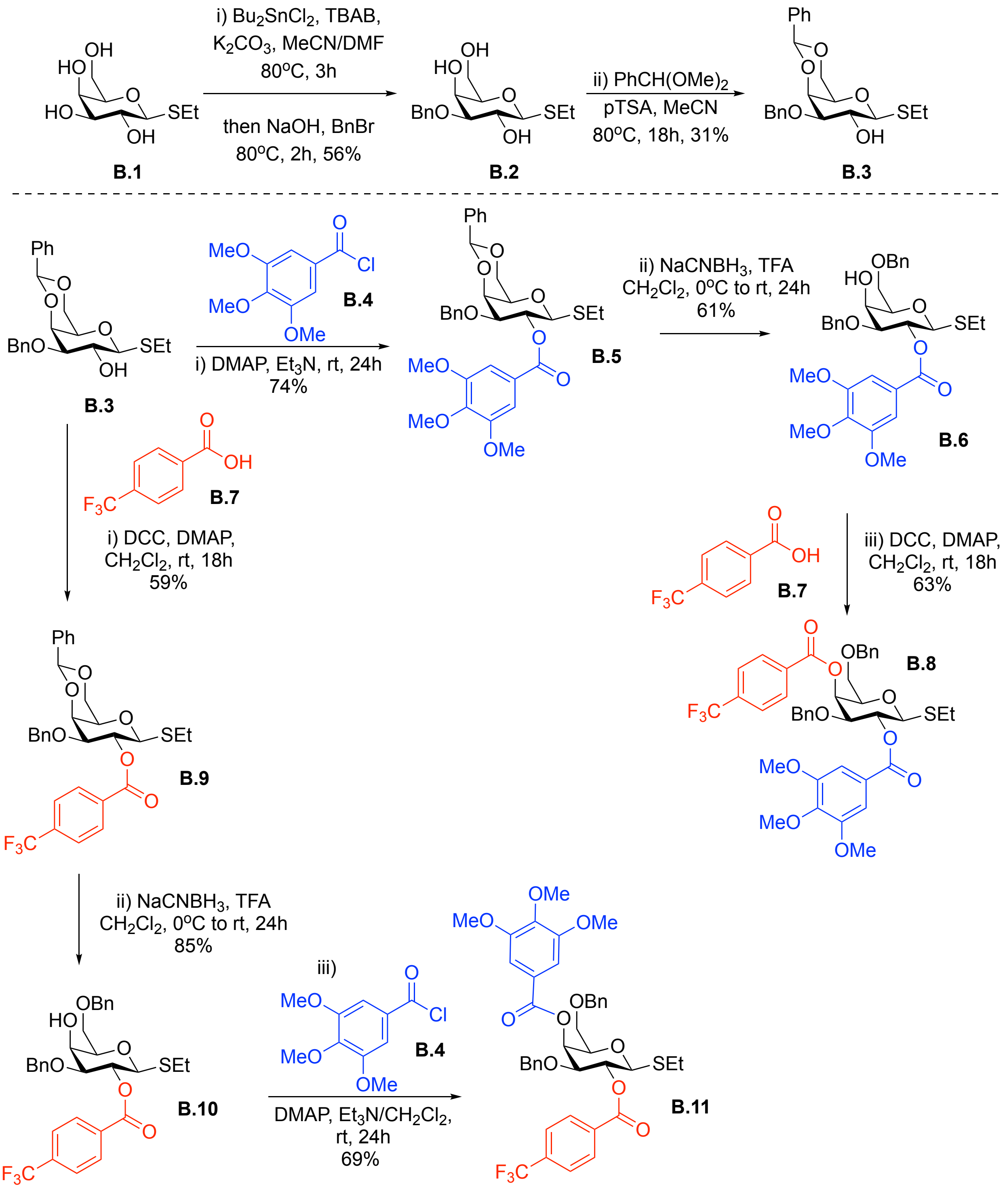

**Synthesis of Substrates in Figure 4 (B.8 and B.11)**

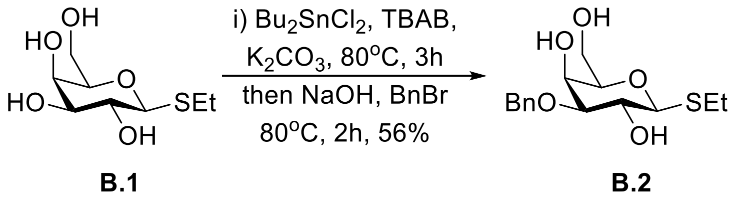


**Ethyl-3-*O*-benzyl-β-D-thiogalactopyranoside (B.2):** Ethyl-β-D-thiogalactopyranoside **B.1** (500 mg, 2.23 mmol, 1.0 eq) was added to a suspension of $Bu_2SnCl_2$ (68 mg, 0.223 mmol, 0.1 eq), $K_2CO_3$ (476 mg, 3.44 mmol, 1.5 eq), benzyl bromide (BnBr, 0.5 3mL, 4.45 mmol, 2.0 eq) and tetrabutylammonium bromide (TBAB, 52 mg, 0.223 mmol, 0.1 eq) in MeCN:DMF (10 mL:1 mL). The reaction was heated to 80 °C and stirred for three hours. Additional BnBr (0.53 mL, 4.45 mmol, 2.0 eq) and NaOH (138 mg, 3.44 mmol, 1.5 eq) was added to the reaction was stirred for an additional two hours at 80 °C. Upon completion determined by TLC, the solvents were removed *in vacuo*, and the crude residue was purified by flash column chromatography (50-75% EtOAc/hexanes) to afford **B.2** (395 mg, 56%) as a white solid.

**HRMS** (ESI-TOF) (m/z): [M+Na]+ calcd for $C_{15}H_{22}O_5S$ 337.1080, found 337.1061.

**$^1$H NMR** (700 MHz, $CDCl_3$) δ 7.47 – 7.31 (m, 5H), 4.83 – 4.70 (m, 2H), 4.31 (d, *J* = 9.7 Hz, 1H), 4.05 (s, 1H), 3.83 (t, *J* = 9.4 Hz, 1H), 3.80 (ddd, *J* = 12.3, 8.6, 4.4 Hz, 1H), 3.54 (t, *J* = 5.6 Hz, 1H), 3.45 (dd, *J* = 9.0, 3.2 Hz, 1H), 2.75 (qq, *J* = 13.5, 7.5 Hz, 2H), 2.59 (d, *J* = 1.9 Hz, 1H), 2.14 (dd, *J* = 8.6, 4.2 Hz, 1H), 1.31 (t, *J* = 7.4 Hz, 3H).

**$^{13}$C NMR** (176 MHz, $CDCl_3$) δ 137.8, 128.8, 128.3, 128.1, 86.4, 81.4, 78.5, 72.4, 69.5, 67.7, 63.0, 24.3, 15.5.

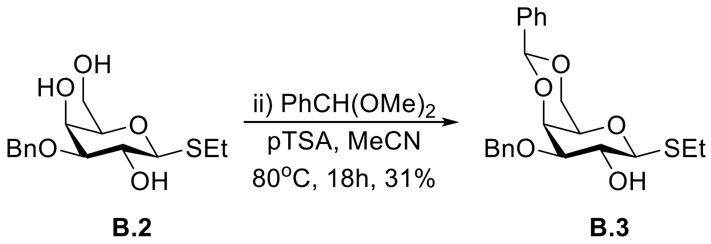


**Ethyl-3-*O*-benzyl-4,6-*O*-benzylidene-β-D-thiogalactopyranoside (B.3):** Benzaldehyde dimethyl acetal (0.36 mL, 2.38 mmol, 1.5 eq) was added dropwise to a solution of **B.2** (500 mg, 1.59 mmol, 1.0eq) and *para*-toluenesulfonic acid (pTSA, 23 mg, 0.119 mmol, 0.075 eq) in MeCN (3.0 mL, 0.5 M). The reaction was heated to reflux (80 °C) and stirred for 18 hours. The reaction was quenched by addition of $Et_3N$ (0.10 mL) and the solvents were removed *in vacuo*. The crude residue was purified by flash column chromatography (10-30% EtOAc/hexanes) to afford **B.3** (197 mg, 31%) as a white solid.

**HRMS** (ESI-TOF) (m/z): [M+Na]+ calcd for $C_{22}H_{26}O_5S$ 425.1393, found 425.1391.

**$^1$H NMR** (400 MHz, $CDCl_3$) δ 7.50 (dd, *J* = 7.3, 2.3 Hz, 2H), 7.45 – 7.27 (m, 8H), 5.45 (s, 1H), 4.78 (d, *J* = 2.4 Hz, 2H), 4.34 (dd, *J* = 14.3, 11.0 Hz, 2H), 4.19 (d, *J* = 3.4 Hz, 1H), 4.07 (t, *J* = 9.4 Hz, 1H), 3.98 (dd, *J* = 12.4, 1.8 Hz, 1H), 3.50 (dd, *J* = 9.3, 3.3 Hz, 1H), 3.42 (s, 1H), 2.91 – 2.70 (m, 2H), 2.53 (s, 1H), 1.33 (t, *J* = 7.5 Hz, 3H).

**$^{13}$C NMR** (101 MHz, cdcl$_3$) δ 138.0, 135.4, 129.2, 128.6, 128.3, 128.0, 128.0, 126.5, 101.4, 85.4, 80.4, 73.7, 71.7, 70.3, 69.6, 68.2, 23.1, 15.4. NMR spectra were in agreement with previously reported data.[1]

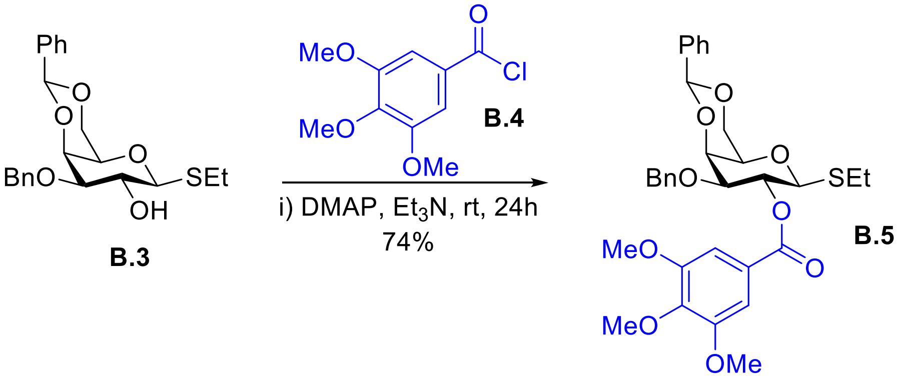


**Ethyl-2-*O*-(3,4,5-trimethoxybenzoyl)-3-*O*-benzyl-4,6-*O*-benzylidene-β-D-thiogalactopyranoside (B.5):** To a solution of **B.3** (46 mg, 0.114 mmol, 1.0 eq) and 4-dimethylaminopyridine (DMAP, 3 mg, 0.023 mmol, 0.2 eq) in $Et_3N$ (0.5 mL) was added 3,4,5-trimethoxybenzoyl chloride **B.4** (106 mg, 0.458 mmol, 4.0 eq) and the reaction was stirred at room temperature for 24 hours. The reaction was quenched by addition of 5mL sat. aq. $NaHCO_3$, and was then extracted with $CH_2Cl_2$ (3x5 mL). The combined organics were dried over $Na_2SO_4$ and concentrated *in vacuo*. The crude residue was purified by flash column chromatography (10-30% EtOAc/hexanes) to afford **B.5** (51 mg, 74%) as a white solid.

**HRMS** (ESI-TOF) (m/z): [M+Na]+ calcd for $C_{32}H_{36}O_9S$ 619.1972, found 619.1961.

**$^1$H NMR** (500 MHz, $CDCl_3$) δ 7.50 (dd, $J$ = 7.6, 2.1 Hz, 2H), 7.42 – 7.26 (m, 10H), 5.45 (s, 1H), 4.77 (d, $J$ = 3.0 Hz, 2H), 4.38 – 4.29 (m, 2H), 4.19 (dd, $J$ = 3.5, 1.1 Hz, 1H), 4.07 (t, $J$ = 9.4 Hz, 1H), 3.96 (s, 1H), 3.92 (s, 9H), 3.50 (dd, $J$ = 9.2, 3.4 Hz, 1H), 3.42 (d, $J$ = 1.4 Hz, 1H), 2.93 – 2.71 (m, 2H), 1.33 (t, $J$ = 7.4 Hz, 3H).

**$^{13}$C NMR** (126 MHz, $CDCl_3$) δ 162.3, 153.2, 143.9, 138.1, 137.8, 129.0, 128.5, 128.2, 127.9, 127.9, 126.4, 123.5, 107.9, 101.3, 85.3, 80.3, 73.6, 71.6, 70.2, 69.5, 68.0, 61.1, 56.4, 22.9, 15.3.

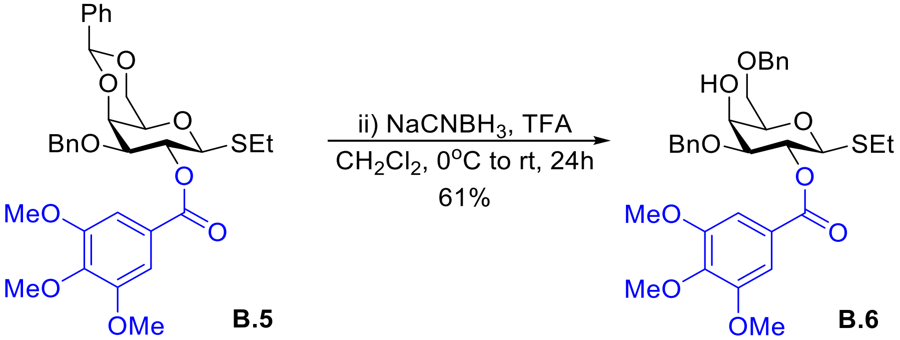


**Ethyl-2-*O*-(3,4,5-trimethoxybenzoyl)-3,6-di-*O*-benzyl-β-D-thiogalactopyranoside (B.6):**

TFA (0.128 mL, 1.68 mmol, 20 eq) was added dropwise to a solution of **B.5** (50 mg, 0.084 mmol, 1.0 eq) and $NaCNBH_3$ (53 mg, 0.838 mmol, 10 eq) in $Et_2O$:$CH_2Cl_2$ (0.3 mL:0.6 mL) at 0 °C. The reaction was stirred for 24 hours while slowly warming to room temperature. The reaction was diluted with EtOAc (5 mL) and diluted with sat. aq. $NaHCO_3$ (10 mL). The organic layer was separated, and the aqueous layer was extracted with EtOAc (2x5 mL). The combined organic layers were washed with brine, then dried over

[1]() Mandal, S. S.; Liao, G.; Guo, Z. *RSC Adv*. **2015**, *5*, 23311.

$Na_2SO_4$ and concentrated *in vacuo*. The crude residue was purified by flash column chromatography (25-50% EtOAc/hexanes) to afford **B.6** (31 mg, 61%) as a white solid.

**HRMS** (ESI-TOF) (m/z): [M+Na]+ calcd for $C_{32}H_{38}O_9S$ 621.2129, found 621.2159.

**$^1$H NMR** (500 MHz, $CDCl_3$) δ 7.30 – 7.08 (m, 12H), 5.44 (t, *J* = 9.7 Hz, 1H), 4.62 (d, *J* = 12.4 Hz, 1H), 4.53 (s, 2H), 4.51 – 4.40 (m, 2H), 4.13 (d, *J* = 3.1 Hz, 1H), 3.85 (s, 3H), 3.82 (s, 6H), 3.79 – 3.67 (m, 2H), 3.65 – 3.53 (m, 2H), 2.72 – 2.59 (m, 2H), 1.16 (t, *J* = 7.4 Hz, 3H).

**$^{13}$C NMR** (126 MHz, $CDCl_3$) δ 165.2, 153.0, 142.6, 138.0, 137.3, 128.6, 128.6, 128.1, 128.0, 128.0, 128.0, 125.0, 107.3, 83.5, 79.2, 73.9, 71.3, 69.8, 69.1, 66.5, 61.0, 56.4, 29.8, 23.8, 15.0.

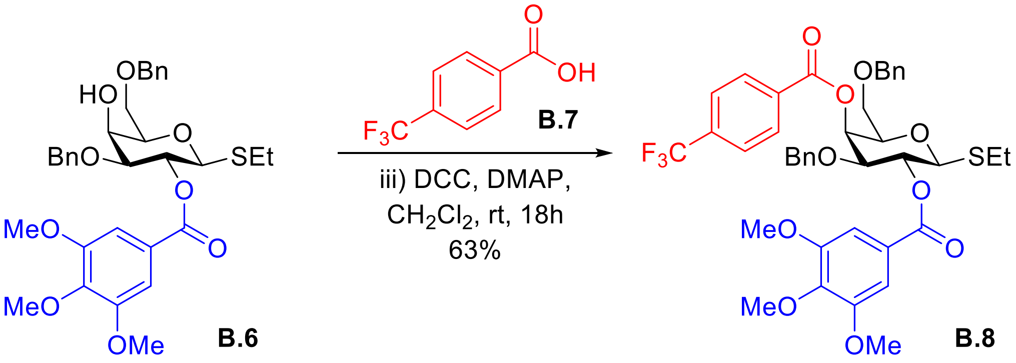


**Ethyl-2-*O*-(3,4,5-trimethoxybenzoyl)-3,6-di-*O*-benzyl-4-*O*-(4-trifluoromethylbenzoyl)-β-D-thiogalactopyranoside (B.8):** DMAP (1.0 mg, 0.01 mmol, 0.2 eq) was added to a solution of 4-(trifluoromethyl)benzoic acid **B.7** (19 mg, 0.10 mmol, 2.0 eq) and dicyclohexylcarbodiimide (DCC, 31 mg, 0.15 mmol, 3.0 eq) in $CH_2Cl_2$ (1.0 mL). The reaction was stirred for one hour at room temperature. **B.6** (30 mg, 0.05 mmol, 1.0 eq) was added in one portion and the reaction was stirred at room temperature for 18 hours. The reaction was filtered through Celite™ and the filtrate was concentrated *in vacuo*. The crude residue was purified by flash column chromatography (20-30% EtOAc) to afford **B.8** (24 mg, 63%) as a white solid.

**HRMS** (ESI-TOF) (m/z): [M+Na]+ calcd for $C_{40}H_{41}O_{10}F_3S$ 793.2265, found 793.2248.

**$^1$H NMR** (500 MHz, $CDCl_3$) δ 8.24 (d, *J* = 8.1 Hz, 2H), 7.78 (d, *J* = 8.2 Hz, 2H), 7.34 – 7.19 (m, 8H), 7.18 – 7.09 (m, 4H), 6.02 (d, *J* = 3.3 Hz, 1H), 5.55 (t, *J* = 9.8 Hz, 1H), 4.75 (d, *J* = 12.8 Hz, 1H), 4.68 (d, *J* = 10.1 Hz, 1H), 4.57 (dd, *J* = 12.3, 9.0 Hz, 2H), 4.47 (d, *J* = 11.8 Hz, 1H), 4.01 (d, *J* = 6.7 Hz, 1H), 3.98 (s, 3H), 3.92 (s, 6H), 3.85 (dd, *J* = 9.6, 3.3 Hz, 1H), 3.72 (dd, *J* = 9.4, 5.6 Hz, 1H), 3.62 (dd, *J* = 9.4, 7.5 Hz, 1H), 2.94 – 2.73 (m, 2H), 1.33 (t, *J* = 7.5 Hz, 3H).

**$^{13}$C NMR** (126 MHz, $CDCl_3$) δ 165.0, 164.6, 152.9, 142.5, 137.3, 137.2, 134.7 (q, *J* = 32.7 Hz), 132.9, 130.4, 128.4, 128.3, 128.1, 128.0, 128.0, 127.9, 127.7, 125.6 (q, *J* = 3.8 Hz), 124.6, 123.8 (q, *J* = 272.3 Hz), 107.2, 83.9, 76.2, 73.8, 70.8, 69.6, 67.7, 67.6, 61.0, 56.3, 24.0, 15.0.

**$^{19}$F NMR** (471 MHz, $CDCl_3$) δ –63.1.

**$[\alpha]_D^{24}$** = + 67.2 (*c* = 0.17, $CH_2Cl_2$).

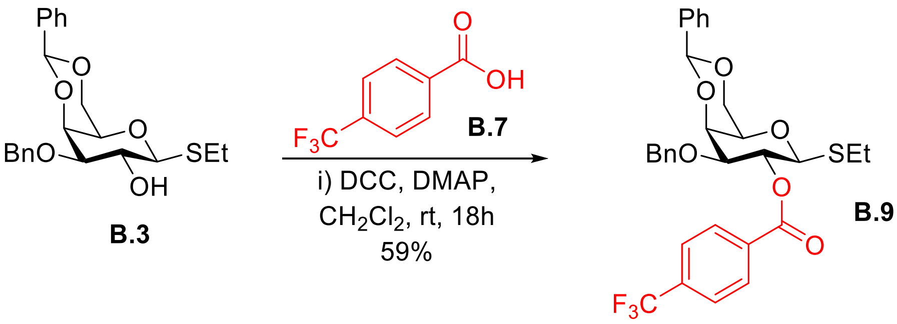


**Ethyl-2-*O*-(4-trifluoromethylbenzoyl)-3-*O*-benzyl-4,6-*O*-benzylidene-β-D-thiogalactopyranoside (B.9):** DMAP (3.0 mg, 0.025 mmol, 0.2 eq) was added to a solution of 4-(trifluoromethyl)benzoic acid **B.7** (47 mg, 0.249 mmol, 2.0 eq) and dicyclohexylcarbodiimide (DCC, 77 mg, 0.373 mmol, 3.0 eq) in $CH_2Cl_2$ (1.0 mL). The reaction was stirred for one hour at room temperature. **B.3** (50 mg, 0.124 mmol, 1.0 eq) was added in one portion and the reaction was stirred at room temperature for 18 hours. The reaction was filtered through Celite™ and the filtrate was concentrated *in vacuo*. The crude residue was purified by flash column chromatography (10-30% EtOAc) to afford **B.9** (42 mg, 59%) as a white solid.

**HRMS** (ESI-TOF) (m/z): [M+Na]+ calcd for $C_{30}H_{29}O_6F_3S$ 597.1529, found 597.1559.

**$^1$H NMR** (500 MHz, $CDCl_3$) δ 8.11 (d, *J* = 8.1 Hz, 2H), 7.72 (d, *J* = 8.2 Hz, 2H), 7.61 – 7.52 (m, 2H), 7.44 – 7.34 (m, 3H), 7.23 – 7.12 (m, 4H), 5.72 (t, *J* = 9.7 Hz, 1H), 5.52 (s, 1H), 4.69 (d, *J* = 12.8 Hz, 1H), 4.59 (d, *J* = 12.8 Hz, 1H), 4.54 (d, *J* = 9.8 Hz, 1H), 4.37 (dd, *J* = 12.4, 1.6 Hz, 1H), 4.30 (d, *J* = 3.7 Hz, 1H), 4.03 (dd, *J* = 12.4, 1.7 Hz, 1H), 3.75 (dd, *J* = 9.6, 3.4 Hz, 1H), 3.51 – 3.47 (m, 1H), 2.91 (dq, *J* = 12.3, 7.5 Hz, 1H), 2.76 (dq, *J* = 12.2, 7.4 Hz, 1H), 1.28 (t, *J* = 7.7 Hz, 3H).

**$^{13}$C NMR** (126 MHz, $CDCl_3$) δ 164.2, 137.8 (d, *J* = 2.0 Hz),, 134.5 (q, *J* = 32.7 Hz), 133.4, 130.3, 129.2, 128.5, 128.4, 128.0, 127.8, 126.6, 125.5 (q, *J* = 3.7 Hz), 123.3 (q, *J* = 273 Hz), 101.5, 82.8, 78.2, 73.4, 71.1, 70.3, 69.5, 69.4, 33.9, 22.9, 15.0.

**$^{19}$F NMR** (471 MHz, $CDCl_3$) δ –63.1.

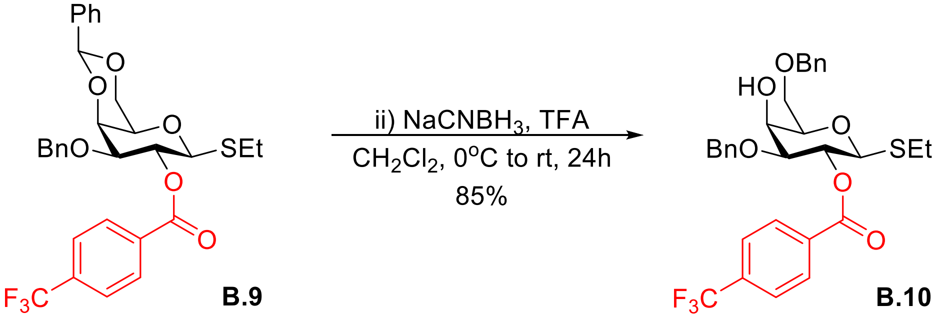


**Ethyl-2-*O*-(4-trifluoromethylbenzoyl)-3,6-di-*O*-benzyl-β-D-thiogalactopyranoside (B.10):** TFA (0.135 mL, 1.4 mmol, 20 eq) was added dropwise to a solution of **B.9** (40 mg, 0.070 mmol, 1.0 eq) and $NaCNBH_3$ (44 mg, 0.70 mmol, 10 eq) in $CH_2Cl_2$ (1.0 mL) at 0 °C. The reaction was stirred for 24 hours while slowly warming to room temperature. The reaction was diluted with EtOAc (5 mL) and diluted with sat. aq. $NaHCO_3$ (10 mL). The organic layer was separated, and the aqueous layer was extracted with EtOAc (2x5 mL). The combined organic layers were washed with brine, then dried over $Na_2SO_4$ and concentrated *in vacuo*. The crude residue was purified by flash column chromatography (5-20% EtOAc/hexanes) to afford **B.10** (34 mg, 85%) as a white solid.

**HRMS** (ESI-TOF) (m/z): [M+Na]+ calcd for $C_{30}H_{31}O_6F_3S$ 599.1686, found 599.1729.

$^{1}$**H NMR** (500 MHz, $CDCl_3$) δ 8.08 (d, *J* = 8.1 Hz, 2H), 7.71 (d, *J* = 8.1 Hz, 2H), 7.40 – 7.28 (m, 5H), 7.22 – 7.04 (m, 5H), 5.52 (t, *J* = 9.6 Hz, 1H), 4.69 (d, *J* = 12.3 Hz, 1H), 4.60 (s, 2H), 4.57 – 4.45 (m, 2H), 4.21 (d, *J* = 3.2 Hz, 1H), 3.83 (dd, *J* = 9.8, 6.3 Hz, 1H), 3.79 (s, 0H), 3.69 (t, *J* = 6.0 Hz, 1H), 3.65 (dd, *J* = 9.3, 3.2 Hz, 1H), 2.81 – 2.65 (m, 2H), 2.64 (d, *J* = 5.8 Hz, 1H), 1.22 – 1.12 (m, 3H).

$^{13}$**C NMR** (126 MHz, $CDCl_3$) δ 164.4, 138.0, 137.2, 134.7 (q, *J* = 32.6 Hz), 133.3, 130.4, 128.6, 128.6, 128.2, 128.0, 128.0, 128.0, 125.5 (q, *J* = 3.7 Hz), 123.8 (q, *J* = 273.3 Hz), 83.4, 79.4, 73.9, 71.4, 70.2, 69.1, 66.4, 29.5, 23.8, 15.0.

$^{19}$**F NMR** (471 MHz, $CDCl_3$) δ –63.1.

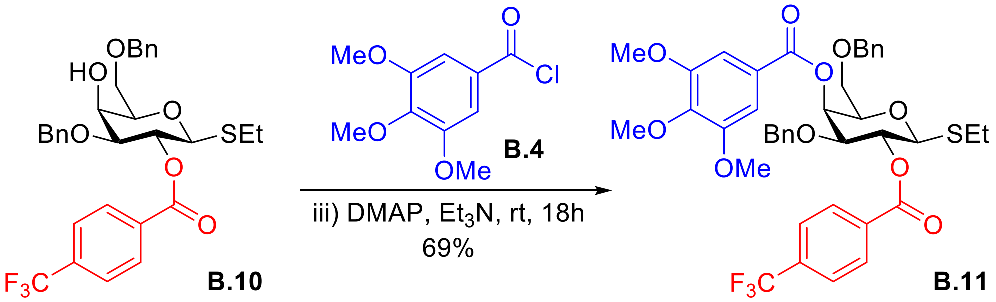


**Ethyl-2-*O*-(4-trifluoromethylbenzoyl)-3,6-di-*O*-benzyl-4-*O*-(3,4,5-trimethoxybenzoyl)-β-D-thiogalactopyranoside (B.11):** To a solution of **B.10** (70 mg, 0.121 mmol, 1.0 eq) and 4-dimethylaminopyridine (DMAP, 55 mg, 0.486 mmol, 4.0 eq) in 1:1 $Et_3N/CH_2Cl_2$ (4.0 mL) was added 3,4,5-trimethoxybenzoyl chloride **B.4** (112 mg, 0.486 mmol, 4.0 eq) and the reaction was stirred at room temperature for 18 hours. The reaction was quenched by addition of 5mL sat. aq. $NaHCO_3$, and was then extracted with $CH_2Cl_2$ (3x5 mL). The combined organics were dried over $Na_2SO_4$ and concentrated *in vacuo*. The crude residue was purified by flash column chromatography (10-30% EtOAc/hexanes) to afford **B.11** (64 mg, 69%) as a white solid.

**HRMS** (ESI-TOF) (m/z): [M+Na]+ calcd for $C_{40}H_{41}O_{10}F_3S$ 793.2265, found 793.2273.

$^{1}$**H NMR** (500 MHz, $CDCl_3$) δ 8.05 (dd, *J* = 8.1, 0.0 Hz, 2H), 7.71 (d, *J* = 8.2 Hz, 2H), 7.37 (s, 2H), 7.33 – 7.22 (m, 4H), 7.19 – 7.15 (m, 1H), 7.14 – 7.03 (m, 4H), 5.94 (d, *J* = 3.1 Hz, 1H), 5.59 (t, *J* = 9.8 Hz, 1H), 4.70 (d, *J* = 12.8 Hz, 1H), 4.61 – 4.40 (m, 4H), 3.94 (d, *J* = 4.0 Hz, 9H), 3.77 (dd, *J* = 9.6, 3.2 Hz, 1H), 3.69 (dd, *J* = 9.5, 5.7 Hz, 1H), 3.59 (dd, *J* = 9.5, 7.2 Hz, 1H), 2.89 – 2.69 (m, 2H), 1.25 (t, *J* = 7.6 Hz, 3H).

$^{13}$**C NMR** (126 MHz, $CDCl_3$) δ 165.6, 164.3, 142.7, 137.6, 137.4, 134.7 (q, *J* = 32.6 Hz), 133.0, 131.3, 130.4, 128.6, 128.4, 128.2, 128.2, 128.0, 127.9, 125.5 (q, *J* = 3.6 Hz), 124.8, 123.8 (q, *J* = 273.5 Hz), 107.3, 83.4, 77.3, 76.5, 73.9, 70.8, 69.8, 68.2, 67.2, 61.1, 56.3, 23.4, 14.9.

$[\alpha]_D^{24}$ = + 104.5 (*c* = 0.67, $CH_2Cl_2$).

**Studies of C2/C4 directed glycosylation of thiogalactosides**

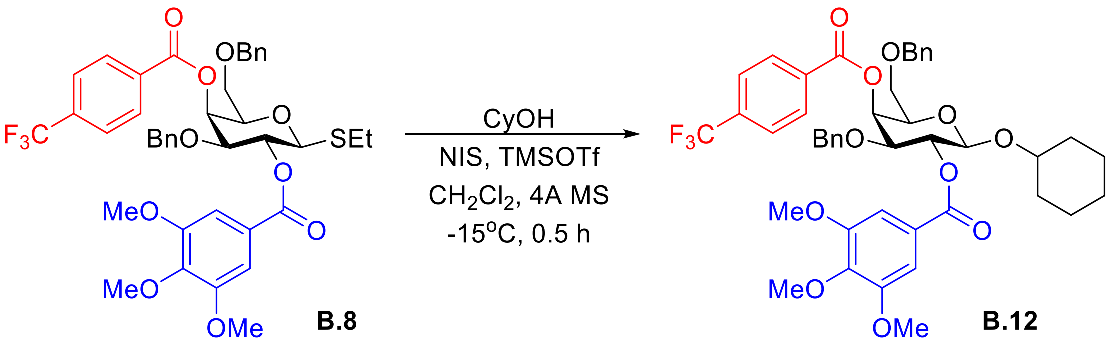

**Glycosylation with C2-EDG,C4-EWG Thioglycloisde B.12:** Thioglycoside **B.8** (10 mg, 0.013 mmol, 1.0 eq) was added to a flame dried flask with freshly activated 4Å molecular sieves (100 mg) and the flask was purged with $N_2$ for five minutes. Anhydrous $CH_2Cl_2$ (0.5 mL) was charged into the flask, and the mixture was stirred for 30 minutes at room temperature. The mixture was cooled to -15 °C (3:1 ice:NaCl) and CyOH (3 μL, 0.026 mmol, 2.0 eq) was added. The mixture was stirred for an additional five minutes, then NIS (4.4 mg, 0.020 mmol, 1.5 eq) and TMSOTf (0.5 μL, 0.003 mmol, 0.2 eq) were added to the reaction at -15 °C under $N_2$ atmosphere. The reaction was stirred for 30 minutes at -15 °C, at which time complete consumption of **B.8** was observed by TLC (30% EtOAc/hexanes) and HRMS. The reaction was quenched with $Et_3N$ (20 μL) at -15 °C, then was warmed to room temperature. The crude reaction was filtered through Celite™, and the filtrate was concentrated in vacuo. The crude residue was dissolved in $CDCl_3$ (0.6 mL) and $CH_2Br_2$ (2 μL, internal standard) was added. The NMR-yield (**B.12,** 79%) and diastereomeric ratio (β-only) were determined by $^1$H-NMR analysis.

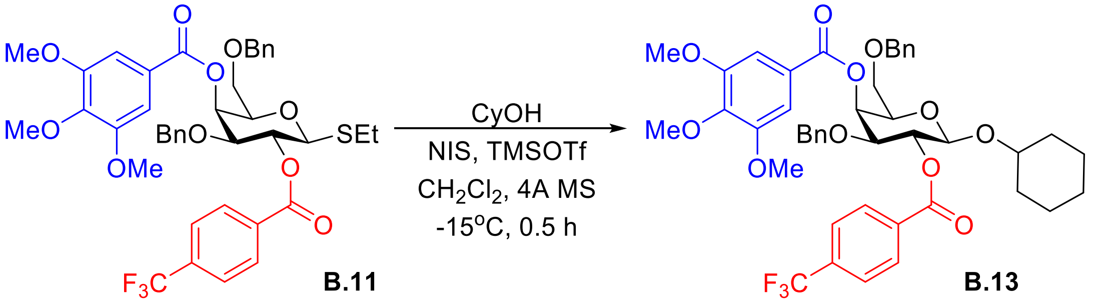

**Glycosylation with C2-EWG,C4-EDG Thioglycloisde B.13:** Thioglycoside **B.11** (0.8 mg, 0.0013 mmol, 1.0 eq) was added to a flame dried flask with freshly activated 4Å molecular sieves (50 mg) and the flask was purged with $N_2$ for five minutes. Anhydrous $CH_2Cl_2$ (0.5 mL) was charged into the flask, and the mixture was stirred for 30 minutes at room temperature. The mixture was cooled to -15 °C (3:1 ice:NaCl) and CyOH (0.3 μL, 0.0026 mmol, 2.0 eq) was added. The mixture was stirred for an additional five minutes, then NIS (0.4 mg, 0.0020 mmol, 1.5 eq) and TMSOTf (0.1 μL, 0.0006 mmol, 0.5 eq) were added to the reaction at -15 °C under $N_2$ atmosphere. The reaction was stirred for 30 minutes at -15 °C, at which time complete consumption of **B.11** was observed by TLC (30% EtOAc/hexanes) and HRMS. The reaction was quenched with $Et_3N$ (20μL) at -15°C, then was warmed to room temperature. The crude reaction was filtered through Celite™, and the filtrate was concentrated in vacuo. The crude residue was dissolved in $CDCl_3$ (0.6 mL) and $CH_2Br_2$ (5 μL, internal standard) was added. The NMR-yield (**B.13**, 67%) and diastereomeric (β -only) ratio were determined by $^1$H-NMR analysis.

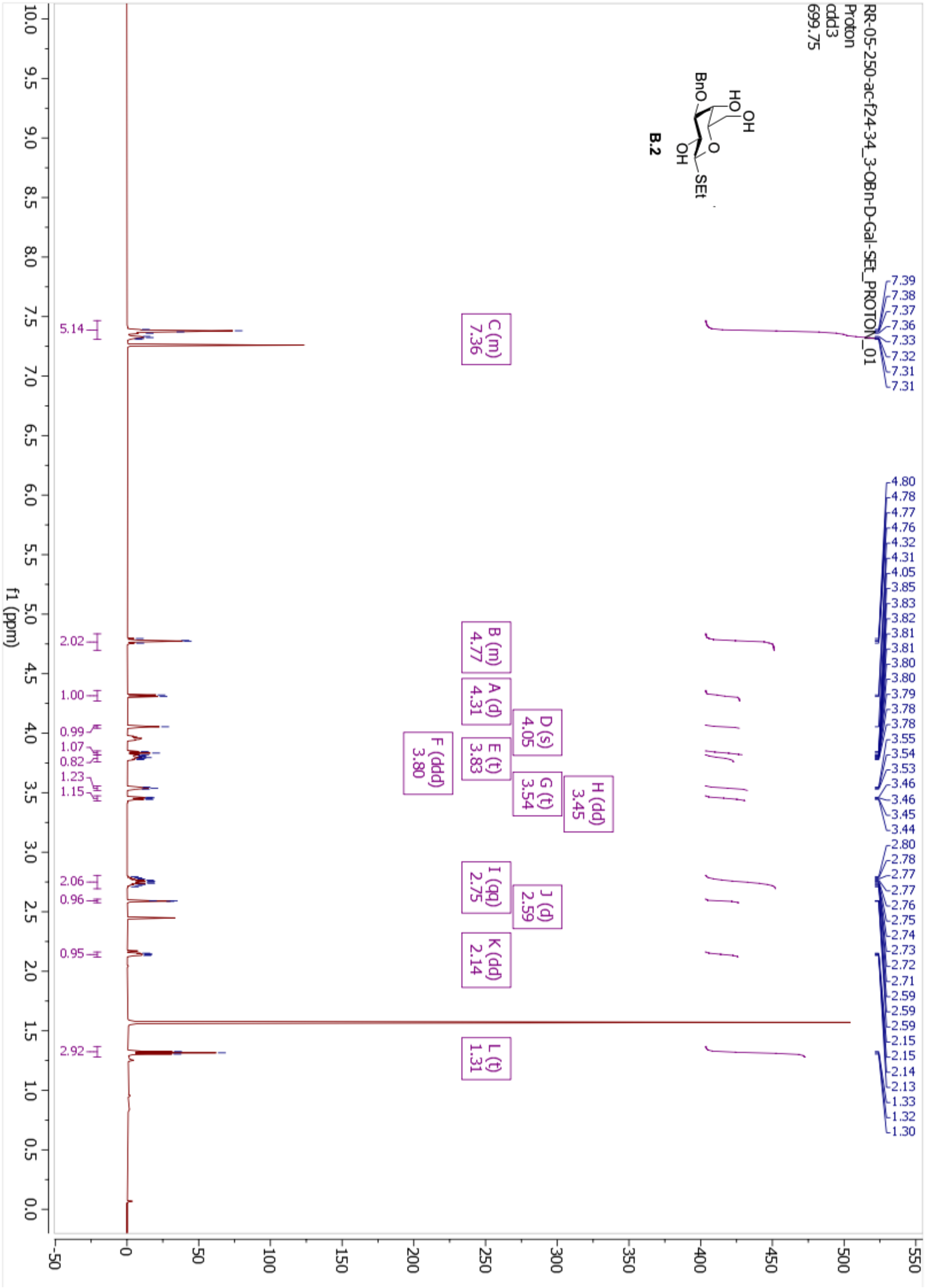
RR-05-250-ac-f24-34_3-OBn-D-Gal-SEt_PROTON_01
Proton
cdcl3
699.75
B.2
C (m) 7.36
B (m) 4.77
A (d) 4.31
D (s) 4.05
E (t) 3.83
F (ddd) 3.80
G (t) 3.54
H (dd) 3.45
I (qq) 2.75
J (d) 2.59
K (dd) 2.14
L (t) 1.31
f1 (ppm)

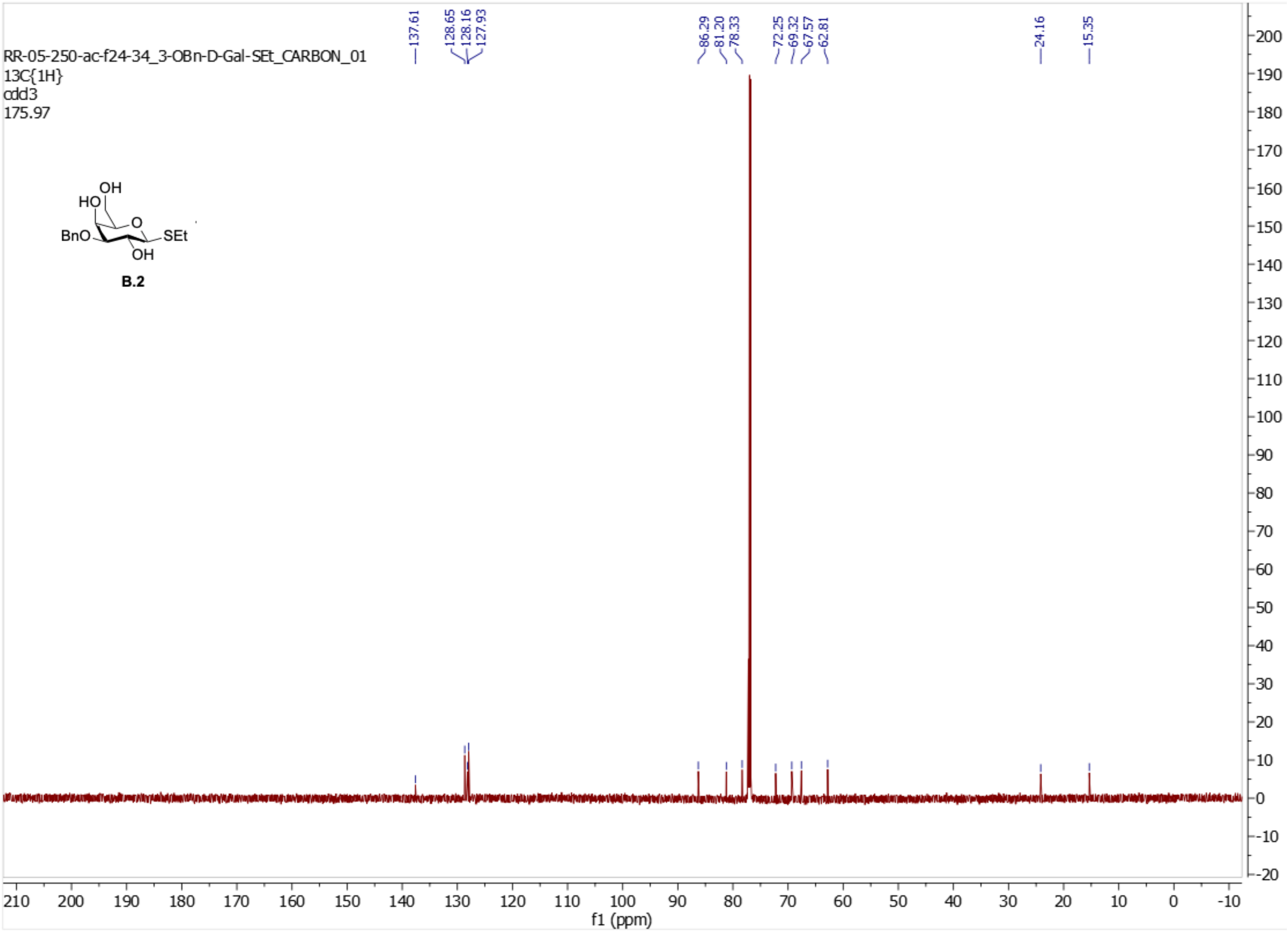
RR-05-250-ac-f24-34_3-OBn-D-Gal-SEt_CARBON_01
13C{1H}
cdcl3
175.97
OH
HO
O
BnO
SEt
OH
B.2
137.61
128.65
128.16
127.93
86.29
81.20
78.33
72.25
69.32
67.57
62.81
24.16
15.35
f1 (ppm)

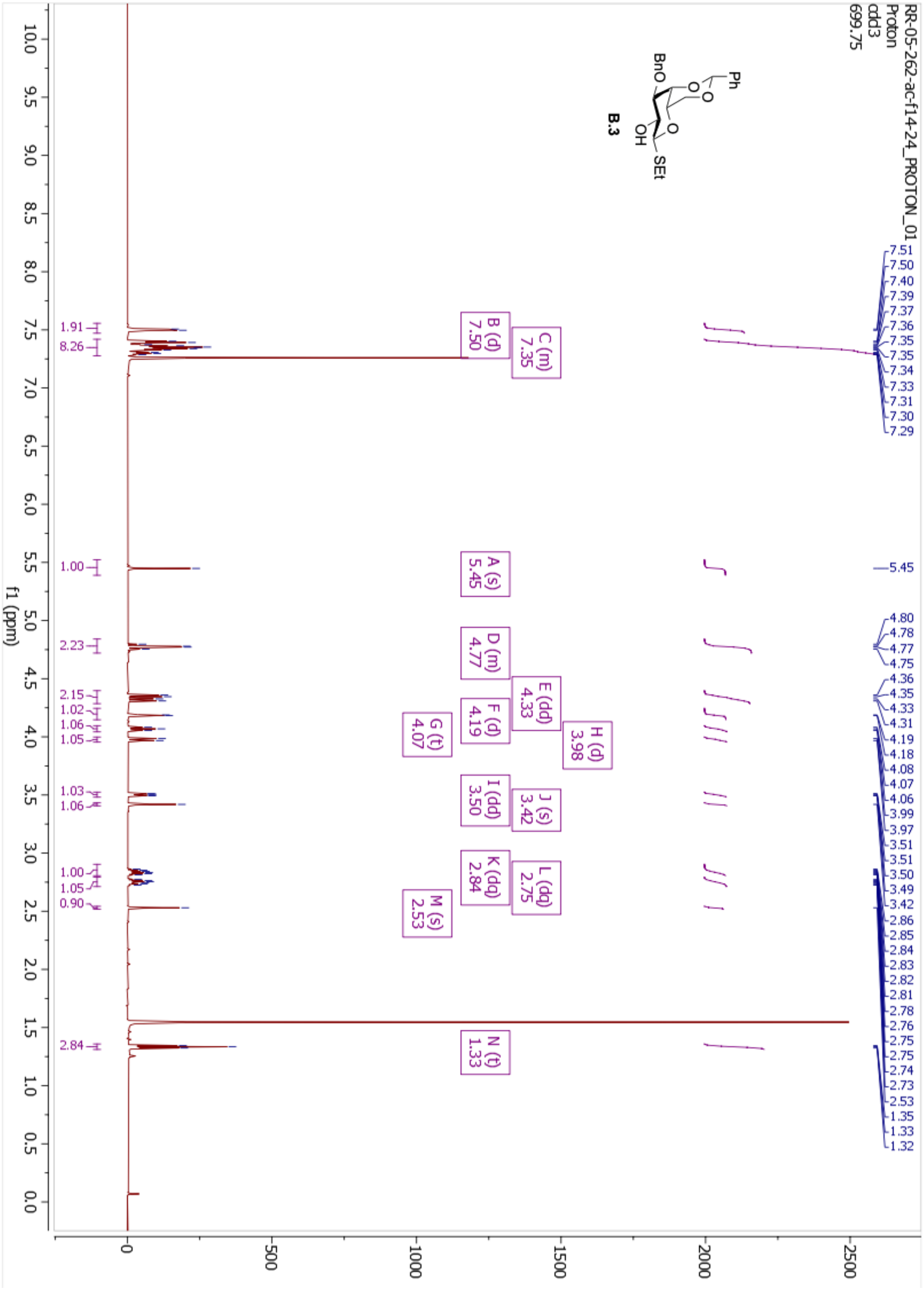

RR-05-262-ac-f14-24_PROTON_01
Proton
cdcl3
699.75
Ph
O
O
O
BnO
OH
SEt
B.3
7.51
7.50
7.40
7.39
7.37
7.36
7.35
7.35
7.34
7.33
7.31
7.30
7.29
5.45
4.80
4.78
4.77
4.75
4.36
4.35
4.33
4.31
4.19
4.18
4.08
4.07
4.06
3.99
3.97
3.51
3.51
3.50
3.49
3.42
2.86
2.85
2.84
2.83
2.82
2.81
2.78
2.76
2.75
2.75
2.74
2.73
2.53
1.35
1.33
1.32
B (d) 7.50
C (m) 7.35
A (s) 5.45
D (m) 4.77
E (dd) 4.33
F (d) 4.19
G (t) 4.07
H (d) 3.98
I (dd) 3.50
J (s) 3.42
K (dq) 2.84
L (dq) 2.75
M (s) 2.53
N (t) 1.33
1.91
8.26
1.00
2.23
2.15
1.02
1.06
1.05
1.03
1.06
1.00
1.05
0.90
2.84
f1 (ppm)

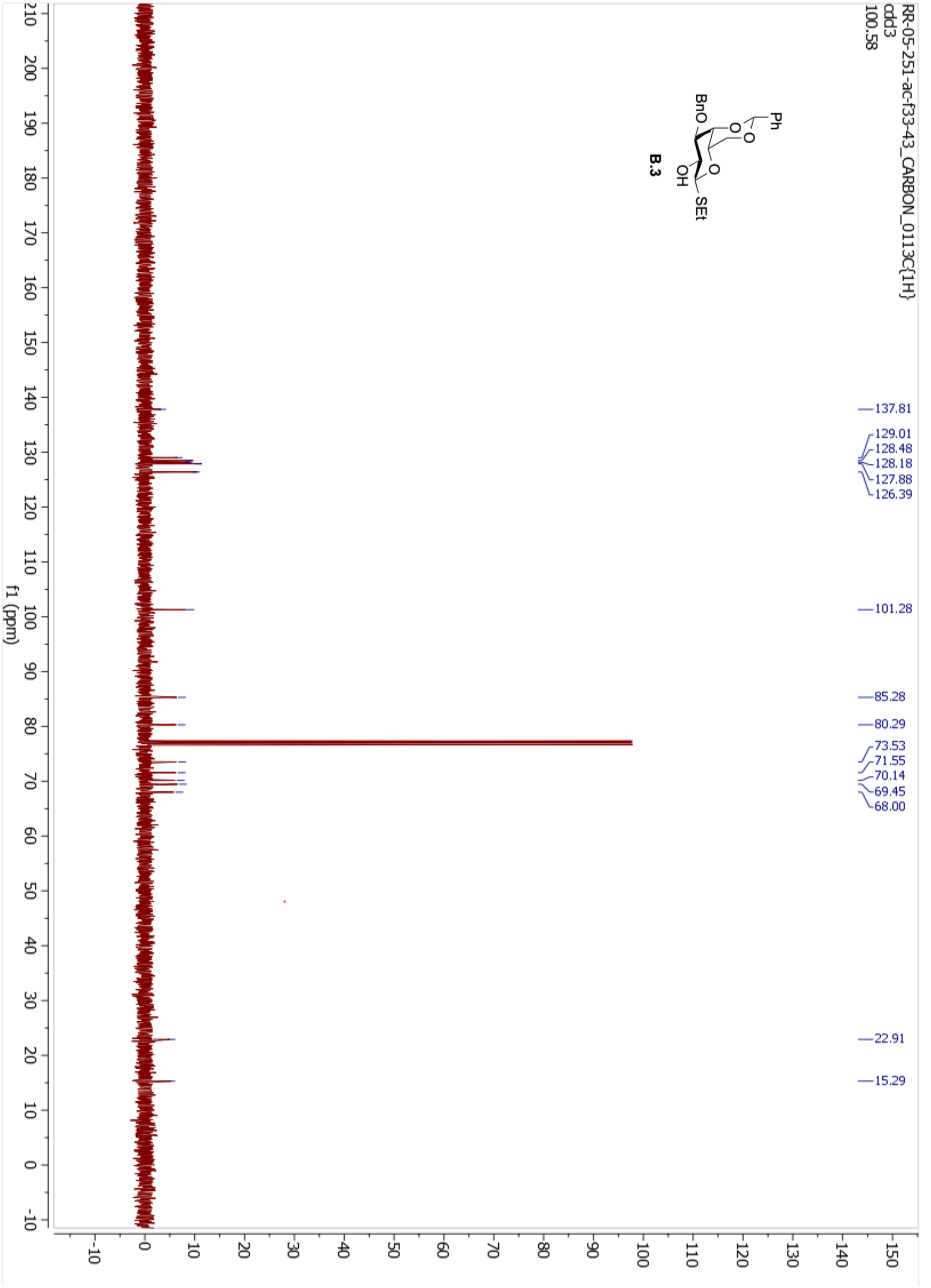

RR-05-251-ac-f33-43_CARBON_0113C{1H}
cdcl3
100.58
Ph
O
O
BnO
O
OH
SEt
B.3
137.81
129.01
128.48
128.18
127.88
126.39
101.28
85.28
80.29
73.53
71.55
70.14
69.45
68.00
22.91
15.29
f1 (ppm)

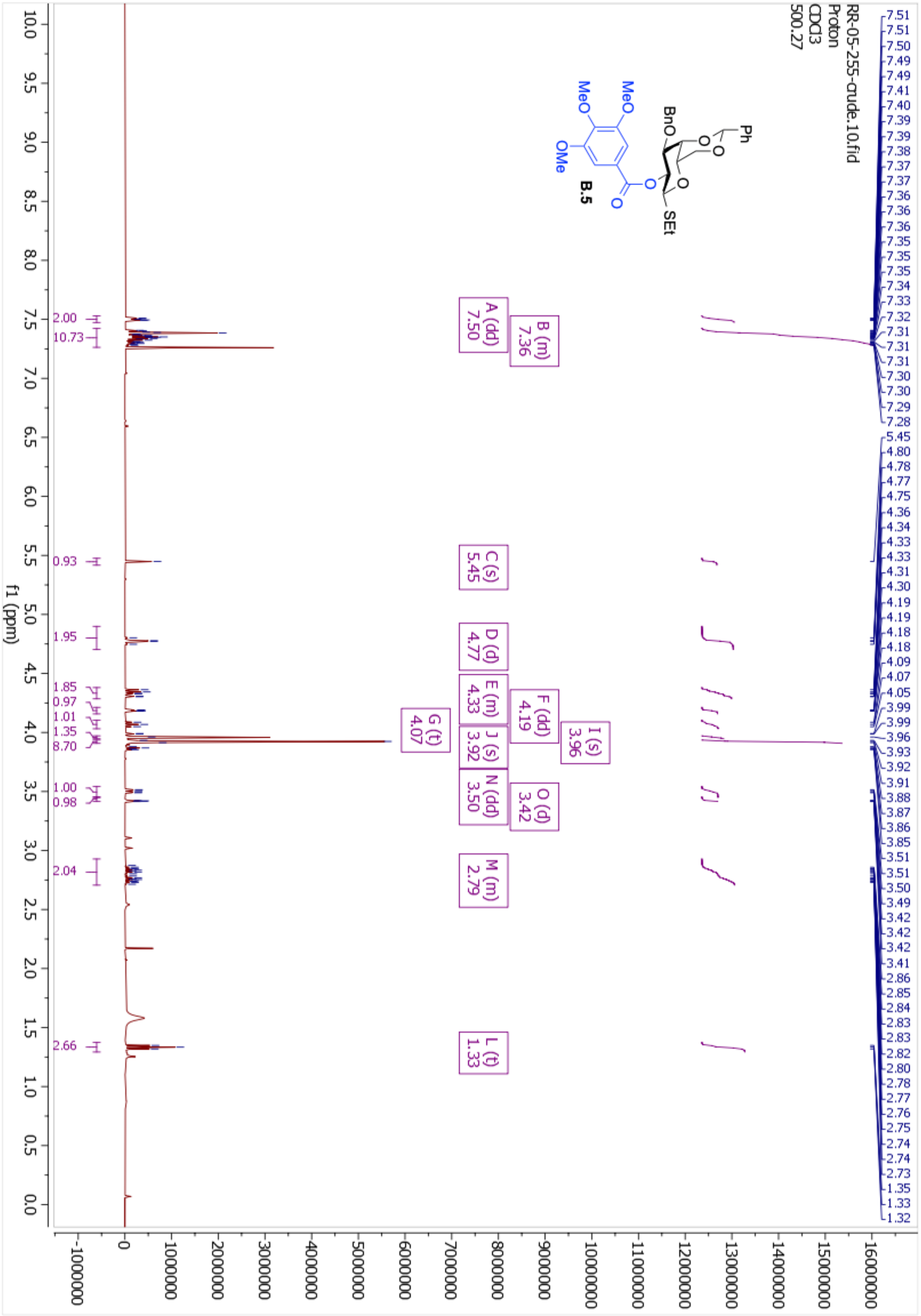
RR-05-255-crude.10.fid
Proton
CDCl3
500.27
B.5
A (dd) 7.50
B (m) 7.36
C (s) 5.45
D (d) 4.77
E (m) 4.33
F (dd) 4.19
G (t) 4.07
I (s) 3.96
J (s) 3.92
N (dd) 3.50
O (d) 3.42
M (m) 2.79
L (t) 1.33
f1 (ppm)

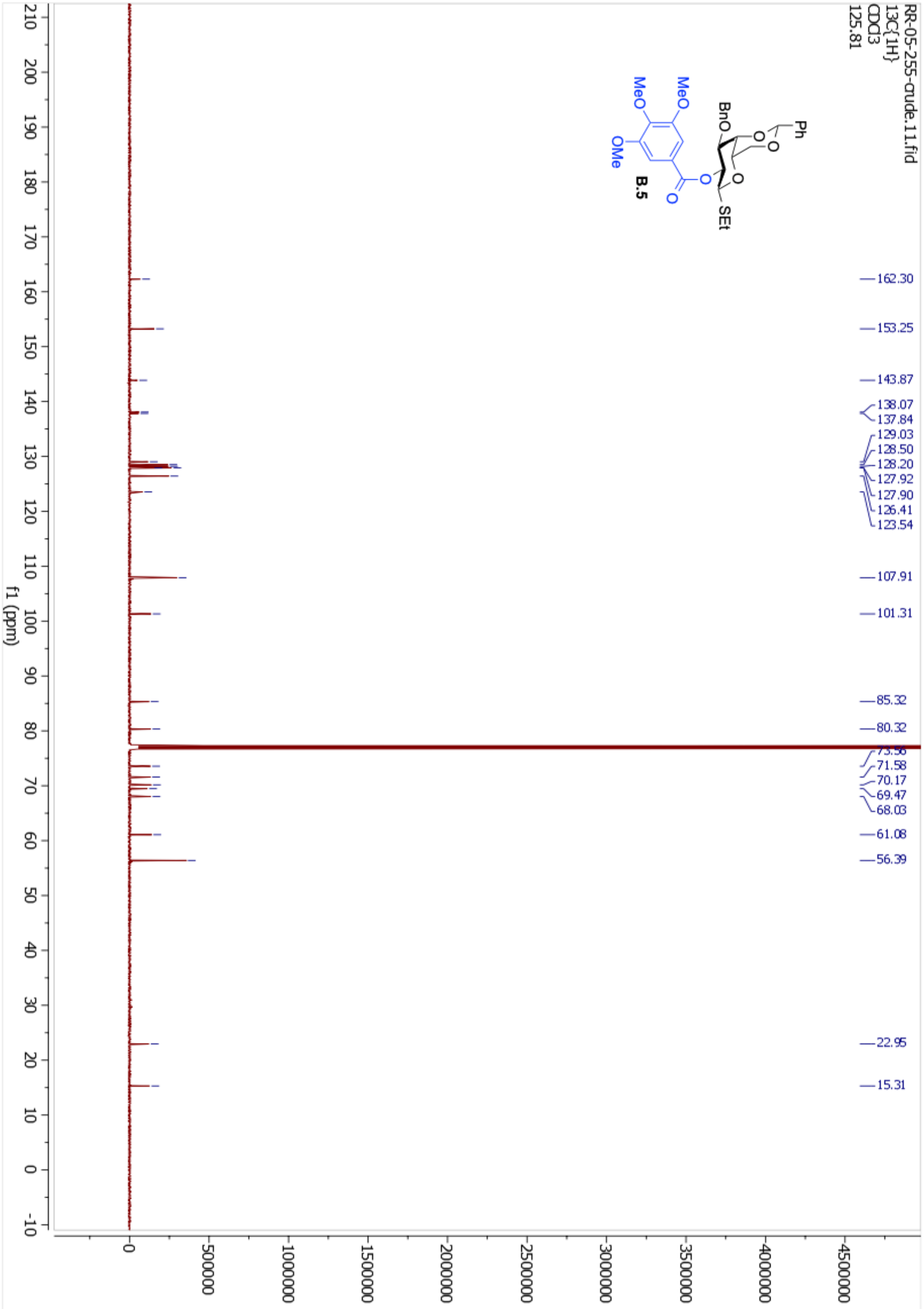

RR-05-255-crude.11.fid
13C{1H}
CDCl3
125.81
Ph
O
O
BnO
O
O
SEt
MeO
MeO
OMe
O
B.5
162.30
153.25
143.87
138.07
137.84
129.03
128.50
128.20
127.92
127.90
126.41
123.54
107.91
101.31
85.32
80.32
71.58
70.17
69.47
68.03
61.08
56.39
22.95
15.31
f1 (ppm)

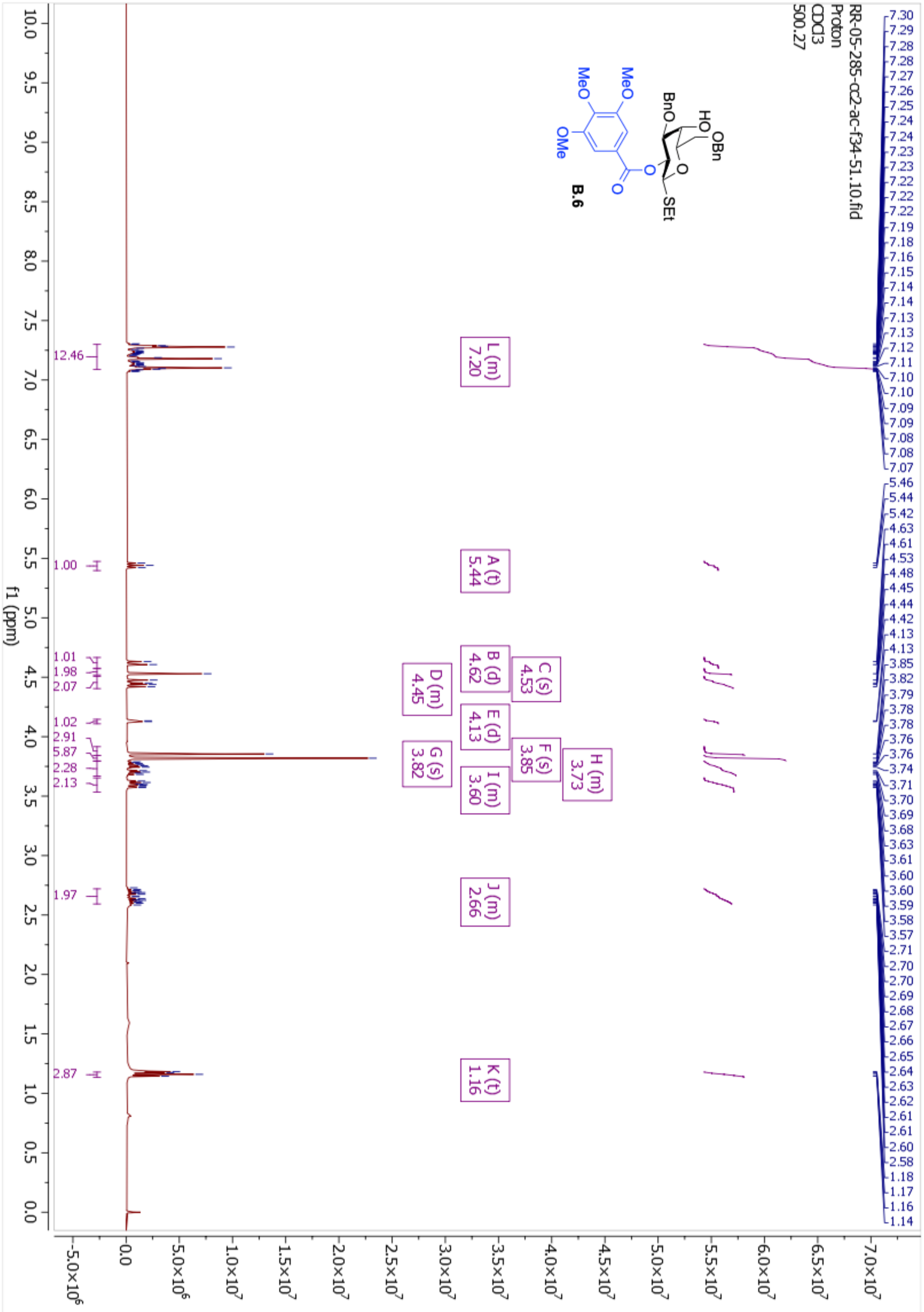

RR-05-285-cc2-ac-f34-51.10.fid
Proton
CDCl3
500.27
B.6
f1 (ppm)

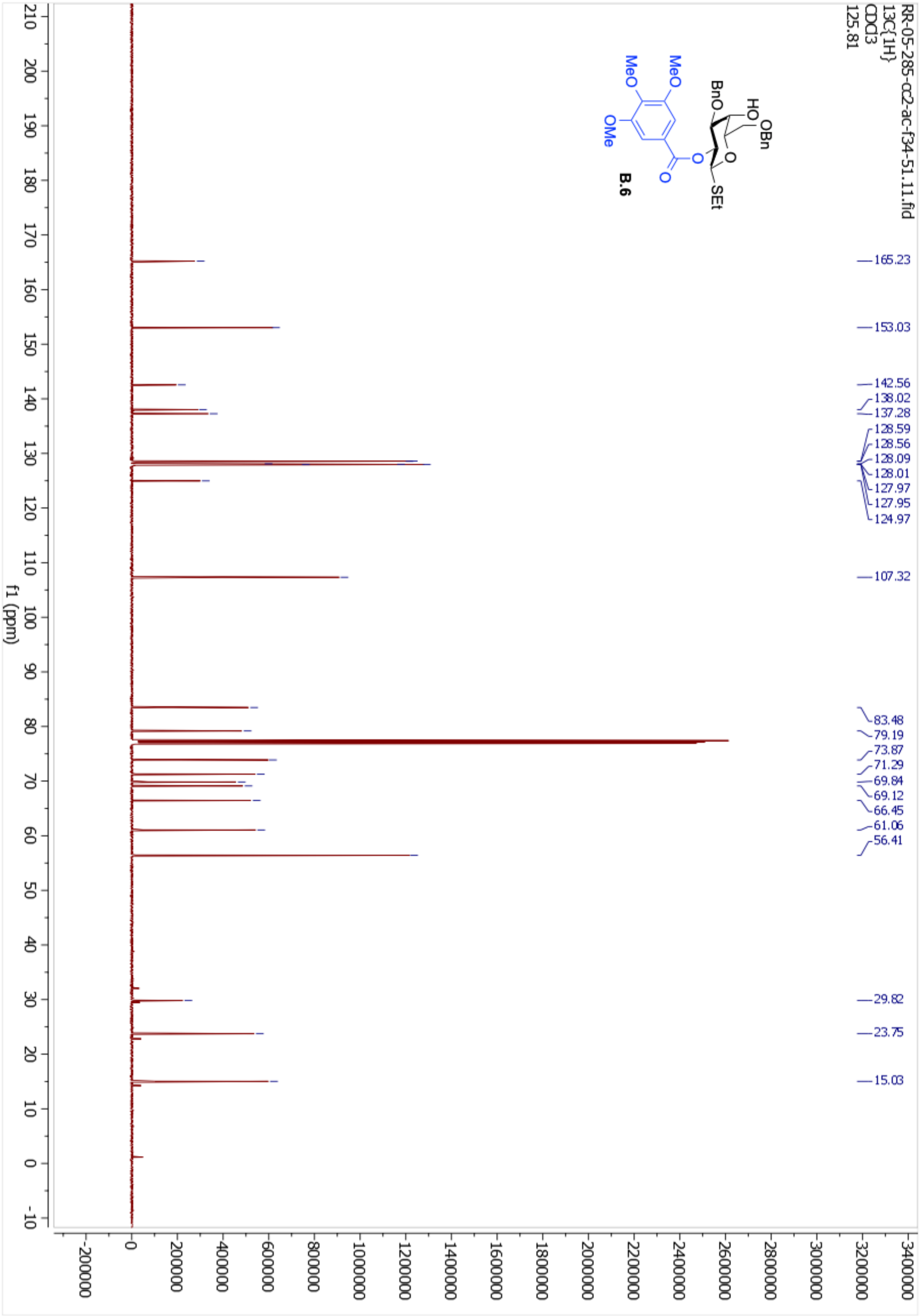
RR-05-285-cc2-ac-f34-51.11.fid
13C{1H}
CDCl3
125.81
OBn
HO
O
BnO
SEt
O
MeO
O
MeO
OMe
B.6
165.23
153.03
142.56
138.02
137.28
128.59
128.56
128.09
128.01
127.97
127.95
124.97
107.32
83.48
79.19
73.87
71.29
69.84
69.12
66.45
61.06
56.41
29.82
23.75
15.03
f1 (ppm)

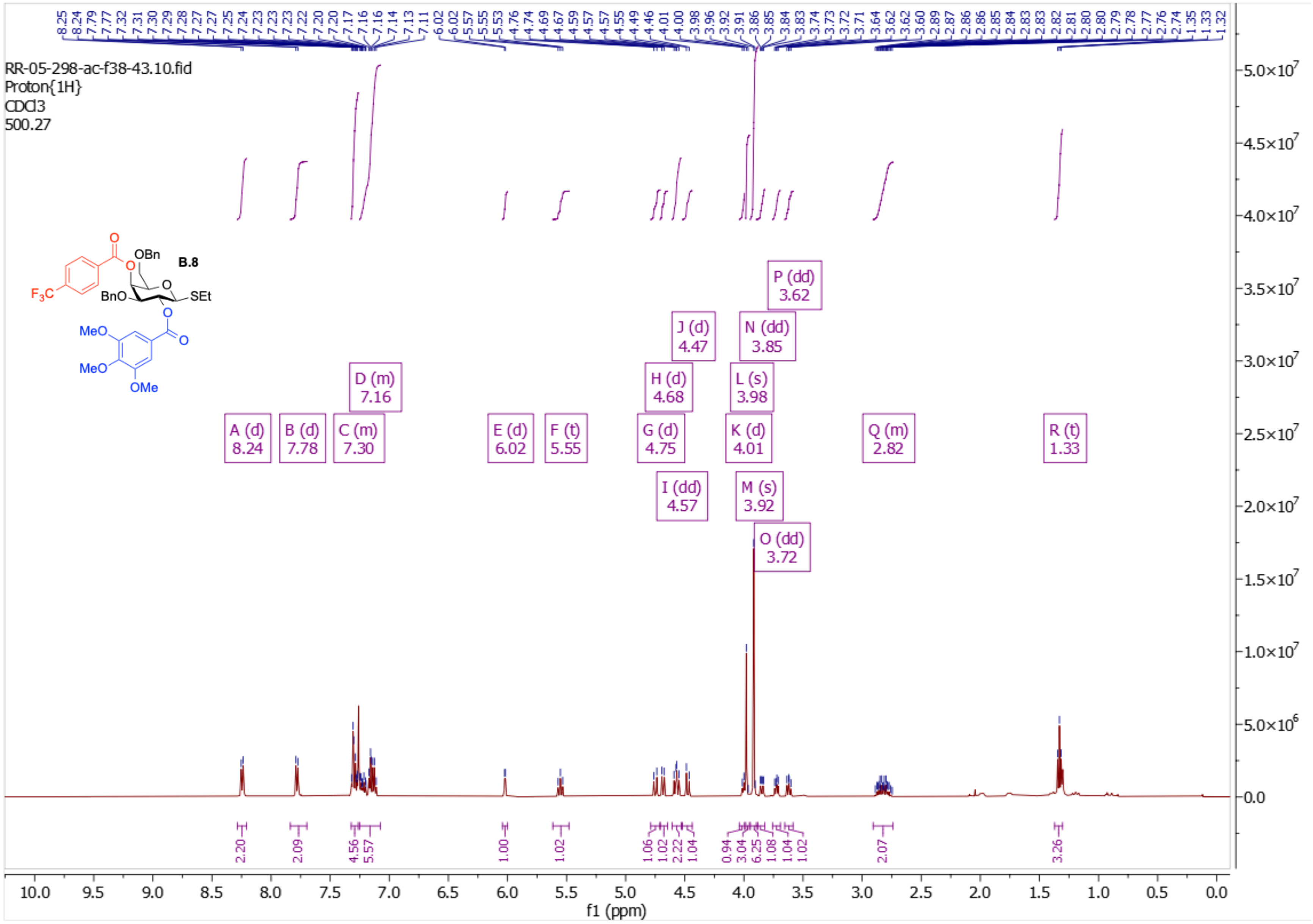

RR-05-298-ac-f38-43.10.fid
Proton{1H}
CDCl3
500.27
B.8
OBn
BnO
SEt
F3C
MeO
MeO
OMe
A (d) 8.24
B (d) 7.78
C (m) 7.30
D (m) 7.16
E (d) 6.02
F (t) 5.55
G (d) 4.75
H (d) 4.68
I (dd) 4.57
J (d) 4.47
K (d) 4.01
L (s) 3.98
M (s) 3.92
N (dd) 3.85
O (dd) 3.72
P (dd) 3.62
Q (m) 2.82
R (t) 1.33
2.20
2.09
4.56
5.57
1.00
1.02
1.06
1.02
2.22
1.04
0.94
3.04
6.25
1.08
1.04
1.02
2.07
3.26
f1 (ppm)

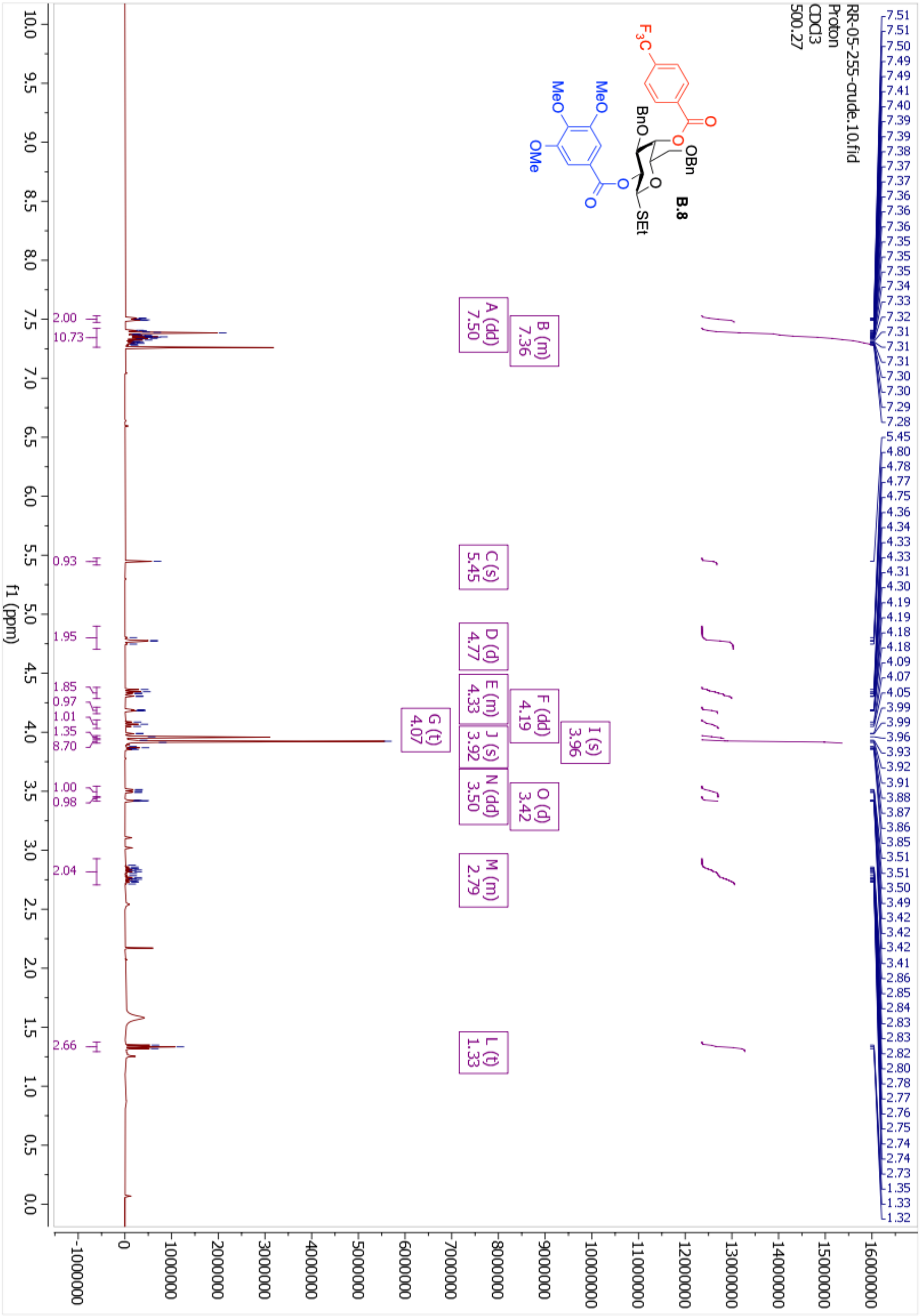
RR-05-255-crude.10.fid
Proton
CDCl3
500.27
B.8
A (dd) 7.50
B (m) 7.36
C (s) 5.45
D (dd) 4.77
E (m) 4.33
F (dd) 4.19
G (t) 4.07
I (s) 3.96
J (s) 3.92
N (dd) 3.50
O (d) 3.42
M (m) 2.79
L (t) 1.33
f1 (ppm)

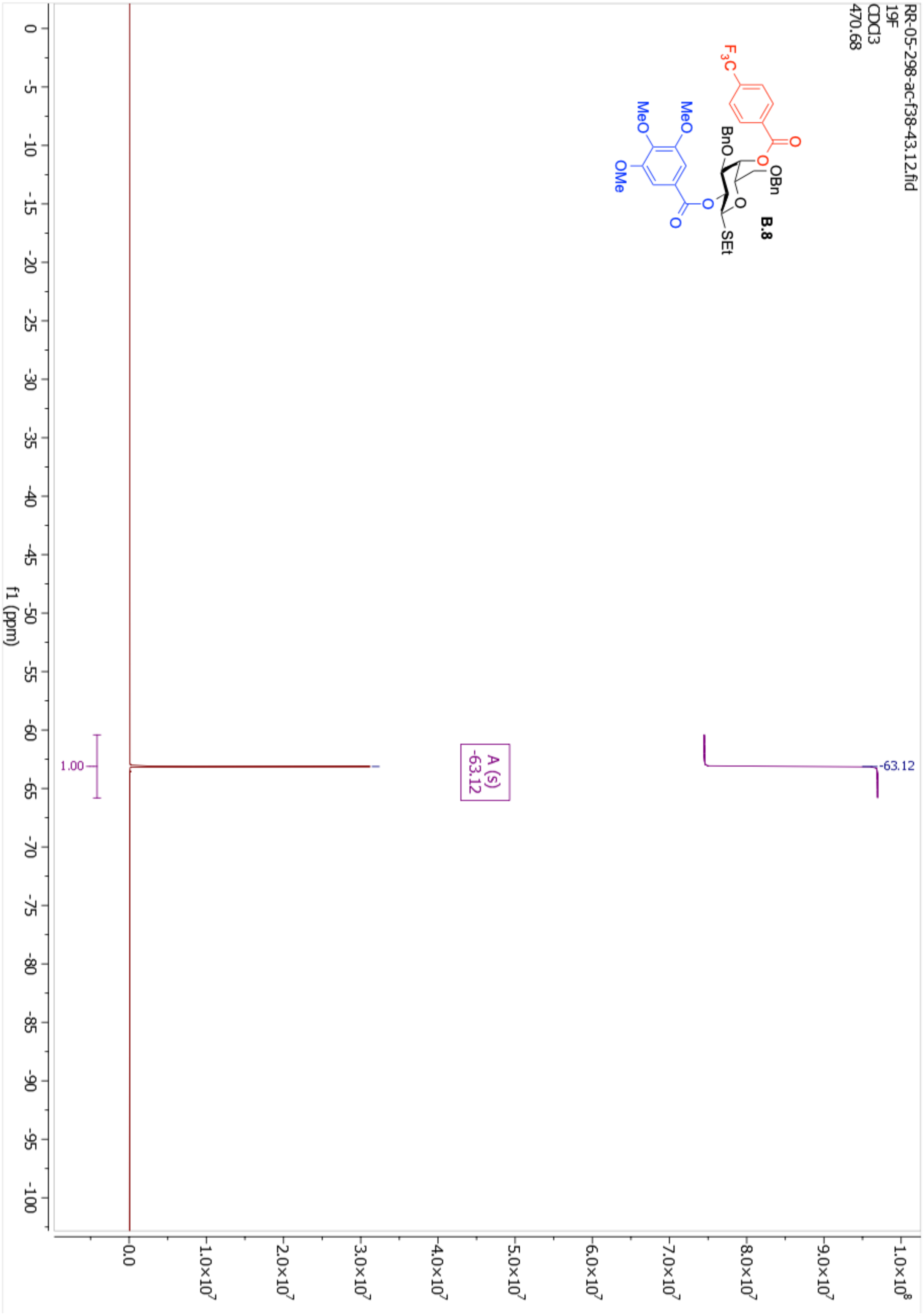
RR-05-298-ac-f38-43.12.fid
19F
CDCl3
470.68
B.8
A (s)
-63.12
-63.12
1.00
f1 (ppm)

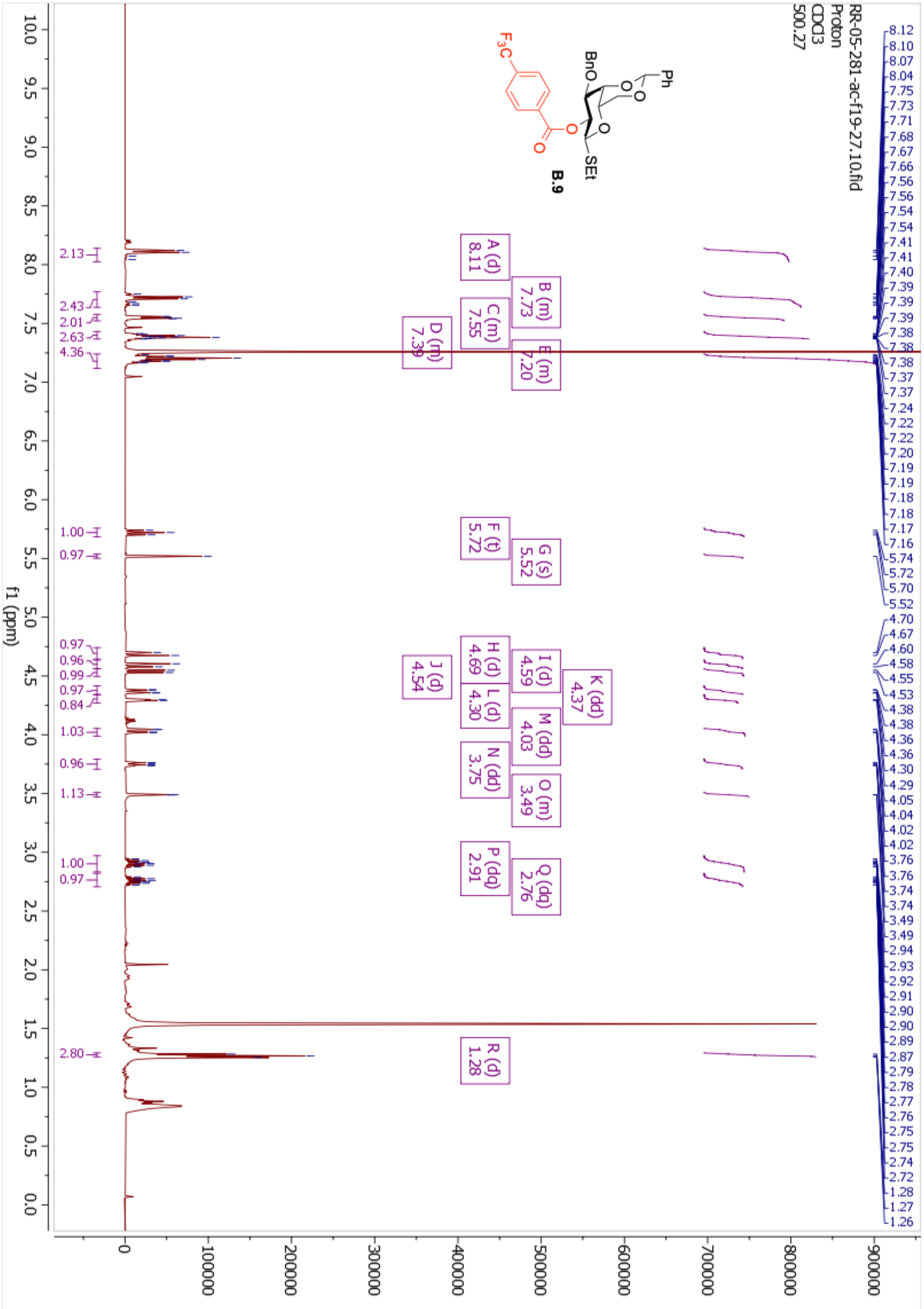
RR-05-281-ac-f19-27.10.fid
Proton
CDCl3
500.27
B.9
Ph
BnO
SEt
F3C
A (d) 8.11
B (m) 7.73
C (m) 7.55
D (m) 7.39
E (m) 7.20
F (t) 5.72
G (s) 5.52
H (d) 4.69
I (d) 4.59
J (d) 4.54
K (dd) 4.37
L (d) 4.30
M (dd) 4.03
N (dd) 3.75
O (m) 3.49
P (dq) 2.91
Q (dq) 2.76
R (d) 1.28
f1 (ppm)

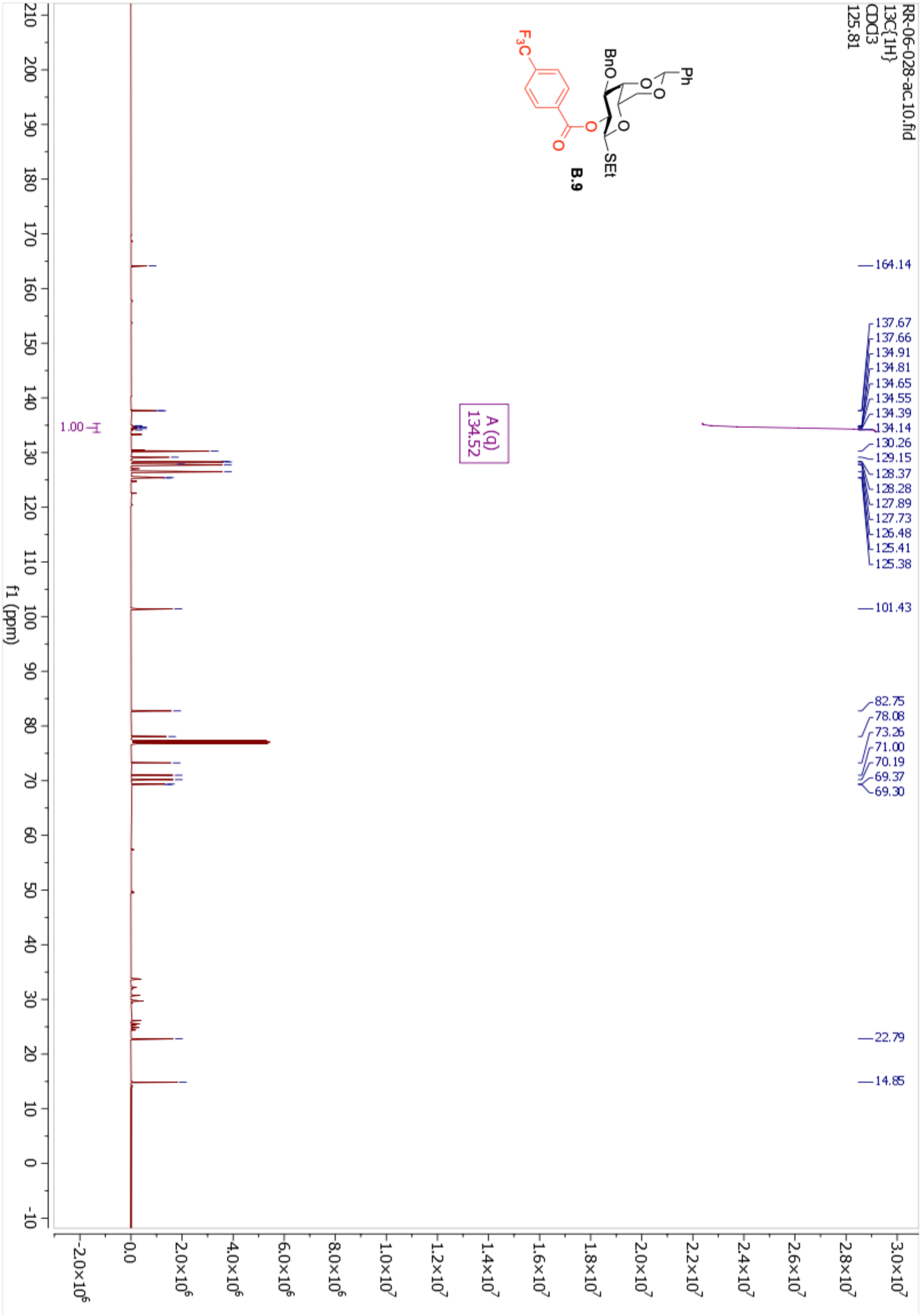
RR-06-028-ac.10.fid
13C{1H}
CDCl3
125.81
B.9
A (q)
134.52
1.00
164.14
137.67
137.66
134.91
134.81
134.65
134.55
134.39
134.14
130.26
129.15
128.37
128.28
127.89
127.73
126.48
125.41
125.38
101.43
82.75
78.08
73.26
71.00
70.19
69.37
69.30
22.79
14.85
f1 (ppm)

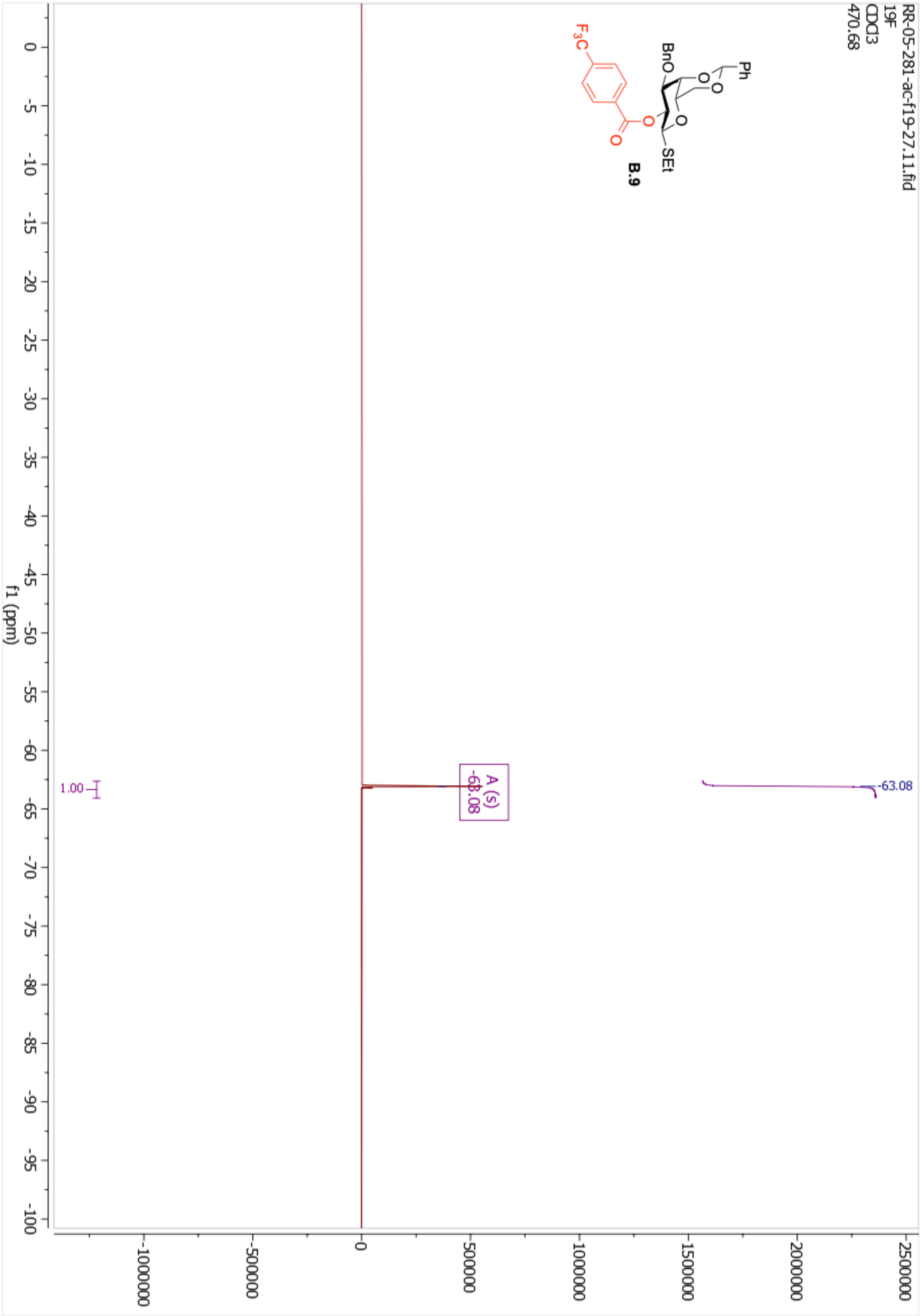
RR-05-281-ac-f19-27.11.fid
19F
CDCl3
470.68
B.9
-63.08
A (s)
-63.08
1.00
f1 (ppm)

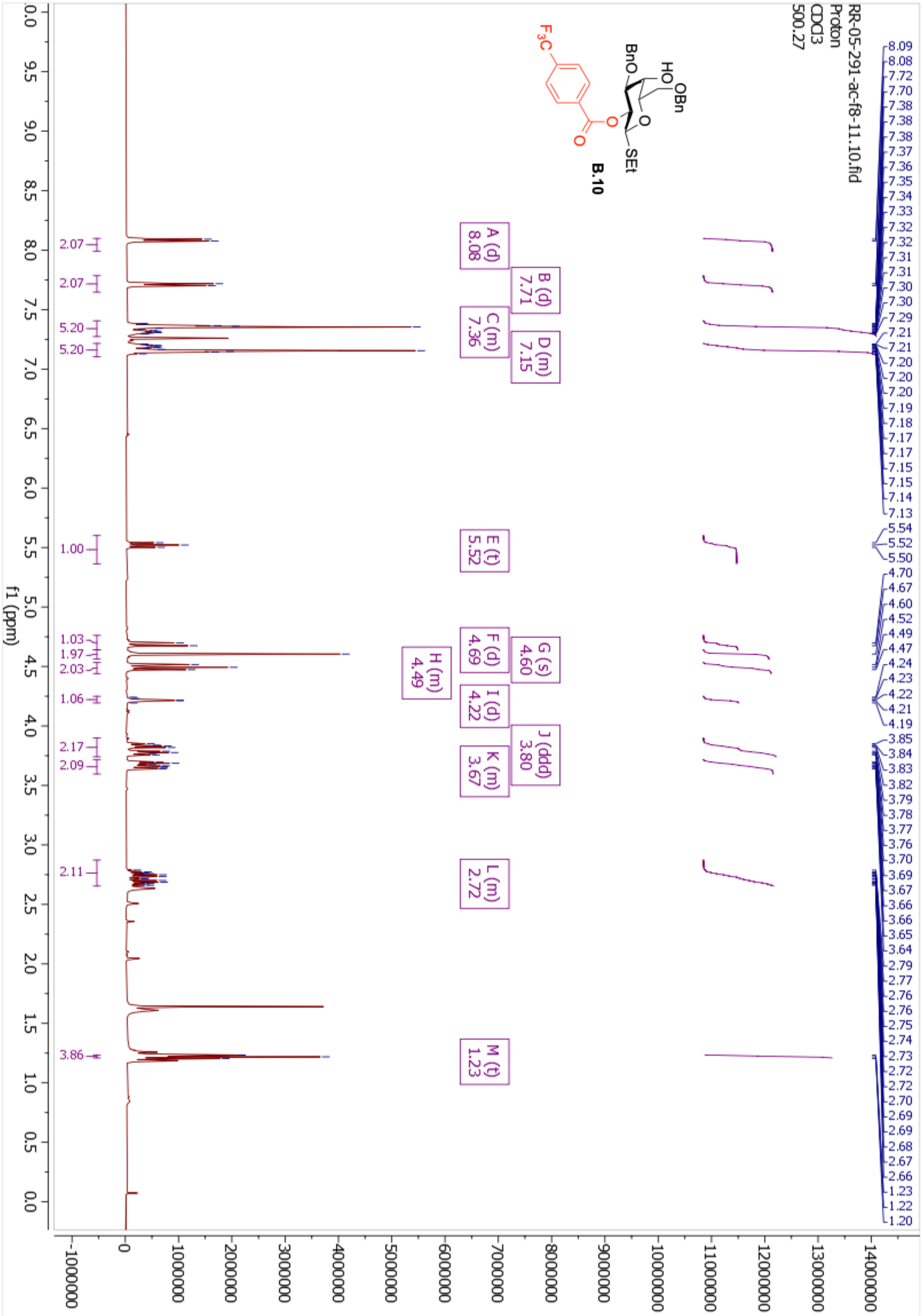
RR-05-291-ac-f8-11.10.fid
Proton
CDCl3
500.27
B.10
A (d) 8.08
B (d) 7.71
C (m) 7.36
D (m) 7.15
E (t) 5.52
F (d) 4.69
G (s) 4.60
H (m) 4.49
I (d) 4.22
J (ddd) 3.80
K (m) 3.67
L (m) 2.72
M (t) 1.23
f1 (ppm)

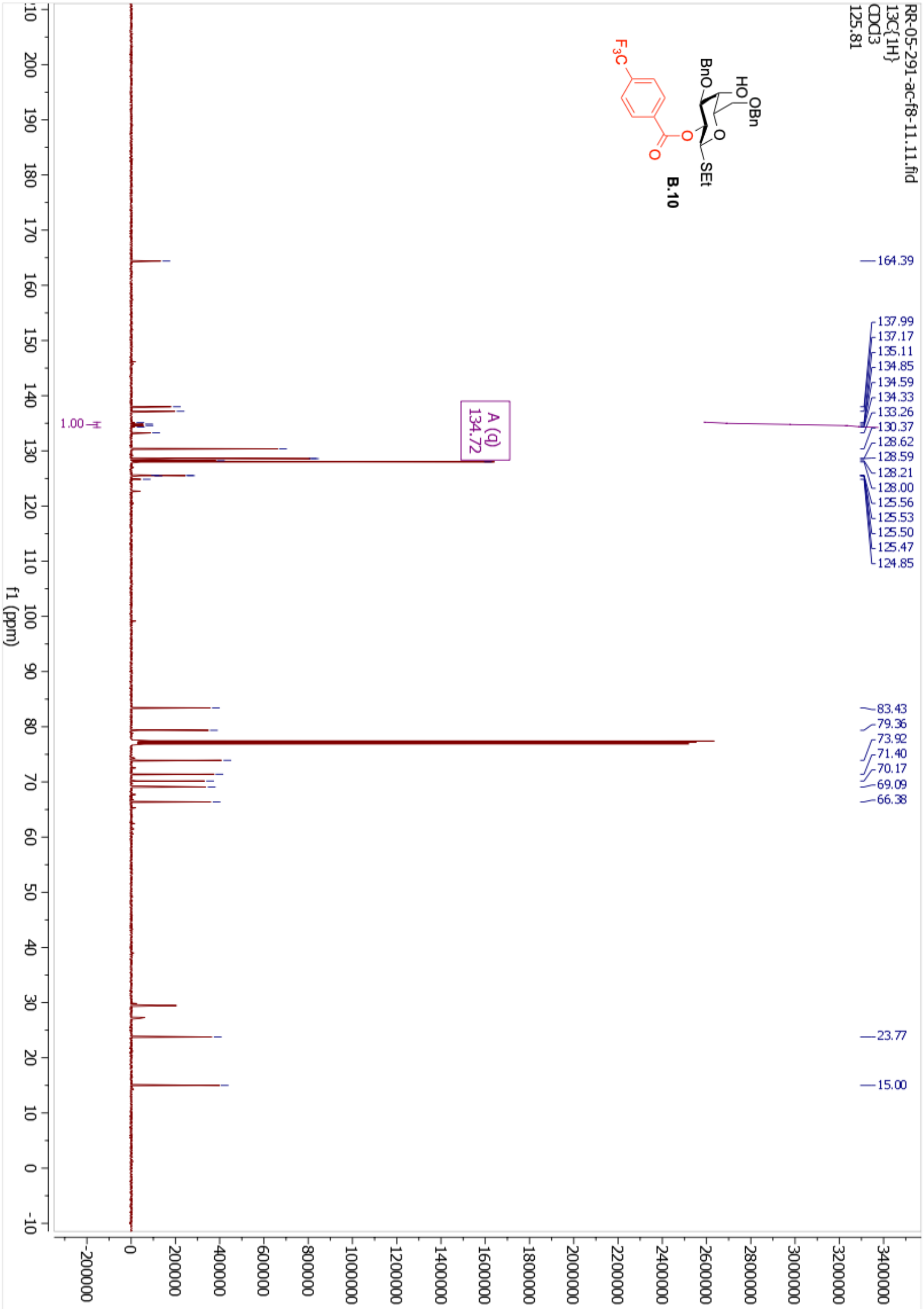
RR-05-291-ac-f8-11.11.fid
13C{1H}
CDCl3
125.81
B.10
164.39
137.99
137.17
135.11
134.85
134.59
134.33
133.26
130.37
128.62
128.59
128.21
128.00
125.56
125.53
125.50
125.47
124.85
A (q)
134.72
1.00
83.43
79.36
73.92
71.40
70.17
69.09
66.38
23.77
15.00
f1 (ppm)

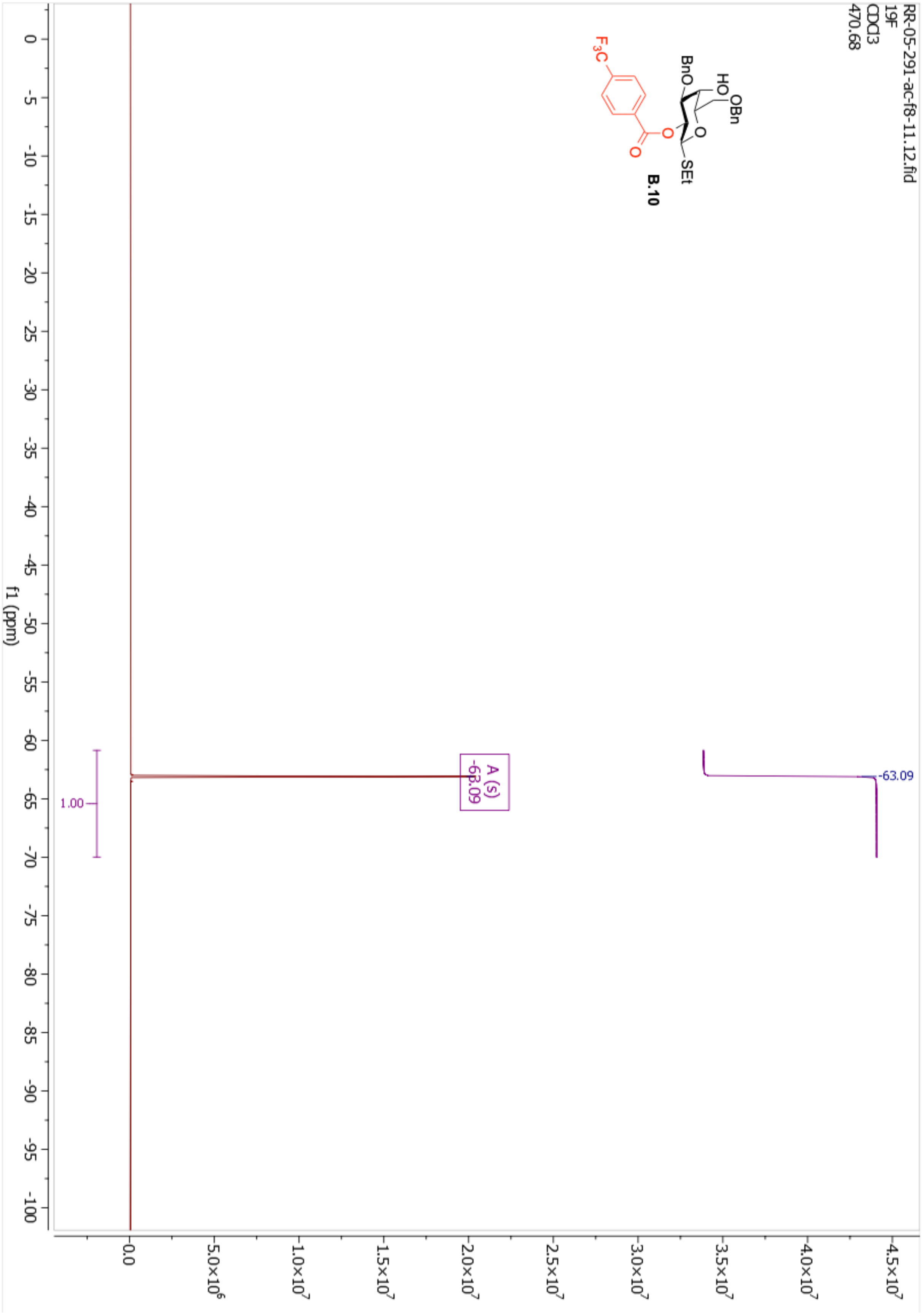
RR-05-291-ac-f8-11.12.fid
19F
CDCl3
470.68
B.10
A (s)
-63.09
-63.09
1.00
f1 (ppm)

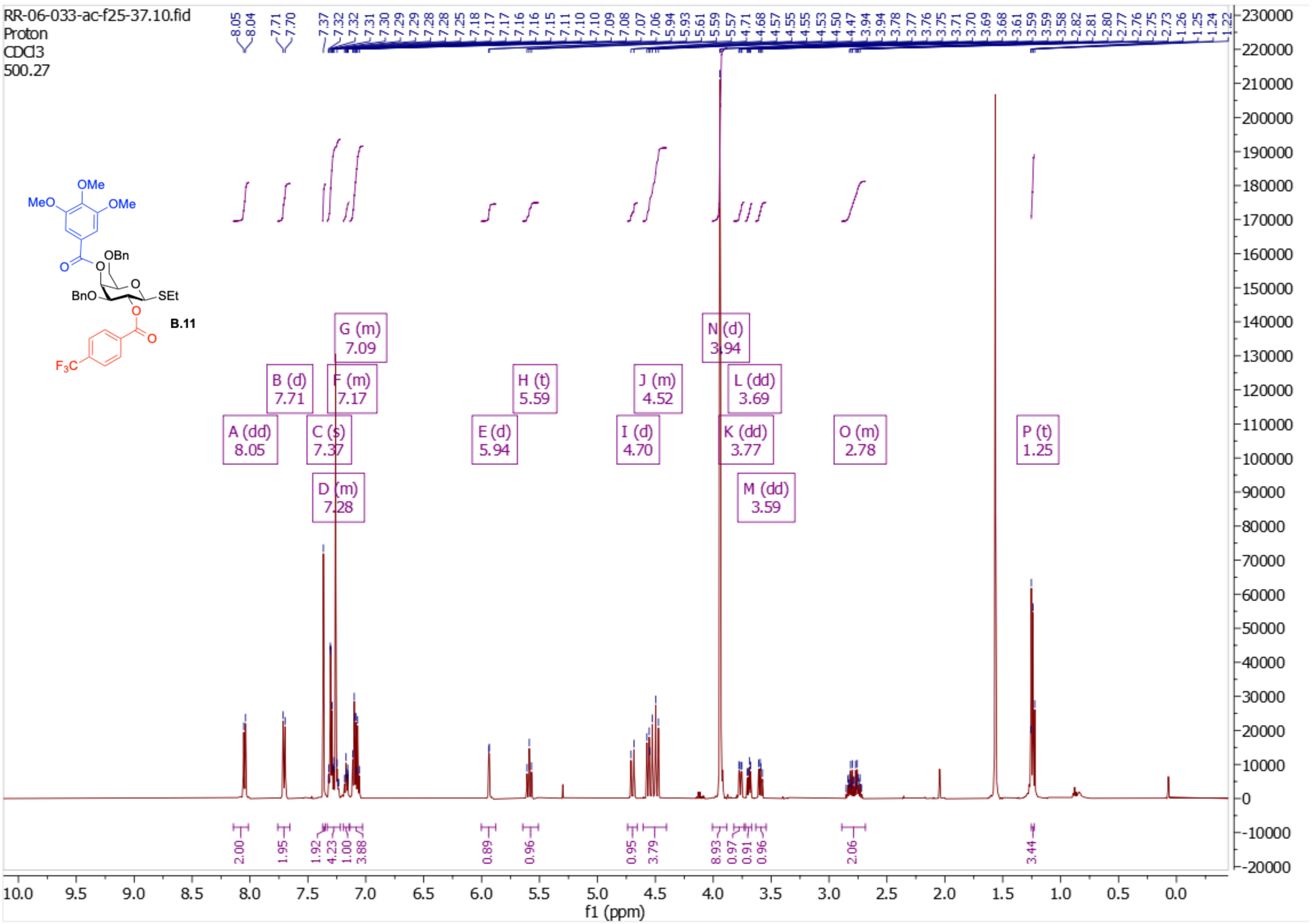
RR-06-033-ac-f25-37.10.fid
Proton
CDCl3
500.27
MeO
OMe
OMe
OBn
BnO
SEt
F3C
B.11
A (dd) 8.05
B (d) 7.71
C (s) 7.37
D (m) 7.28
F (m) 7.17
G (m) 7.09
E (d) 5.94
H (t) 5.59
I (d) 4.70
J (m) 4.52
N (d) 3.94
K (dd) 3.77
L (dd) 3.69
M (dd) 3.59
O (m) 2.78
P (t) 1.25
2.00
1.95
1.92
4.23
1.00
3.88
0.89
0.96
0.95
3.79
8.93
0.97
0.91
0.96
2.06
3.44
8.05 8.04 7.71 7.70 7.37 7.32 7.32 7.31 7.30 7.29 7.29 7.28 7.28 7.25 7.18 7.17 7.17 7.16 7.16 7.15 7.11 7.10 7.10 7.09 7.08 7.07 7.06 5.94 5.93 5.61 5.59 5.57 4.71 4.68 4.57 4.55 4.55 4.53 4.50 4.47 3.94 3.94 3.78 3.77 3.76 3.75 3.71 3.70 3.69 3.68 3.61 3.59 3.59 3.58 2.82 2.81 2.80 2.77 2.76 2.75 2.73 1.26 1.25 1.24 1.22
f1 (ppm)

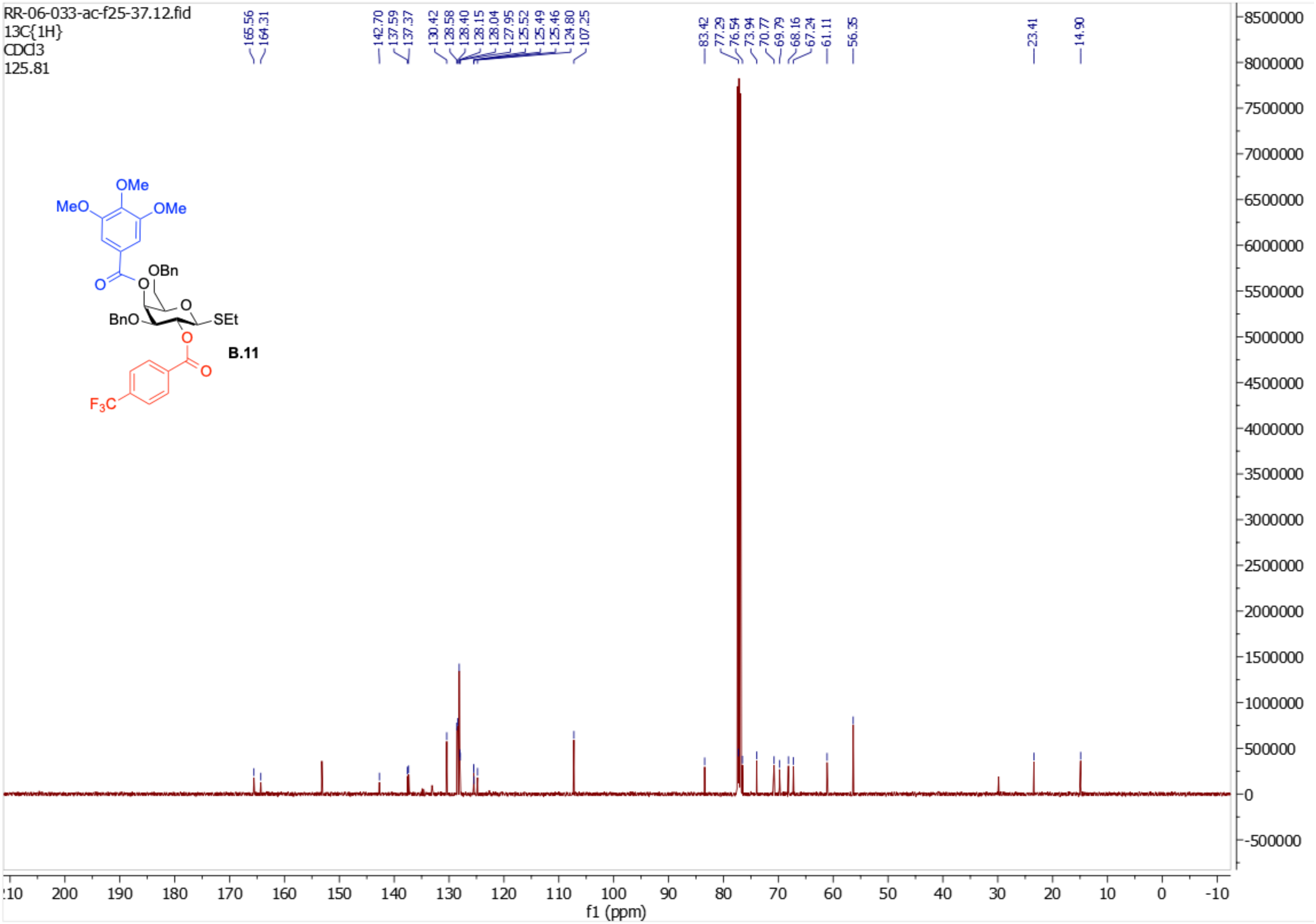
RR-06-033-ac-f25-37.12.fid
13C{1H}
CDCl3
125.81
165.56
164.31
142.70
137.59
137.37
130.42
128.58
128.40
128.15
128.04
127.95
125.52
125.49
125.46
124.80
107.25
83.42
77.29
76.54
73.94
70.77
69.79
68.16
67.24
61.11
56.35
23.41
14.90
OMe
MeO
OMe
OBn
O
O
BnO
O
SEt
O
O
F3C
B.11
f1 (ppm)

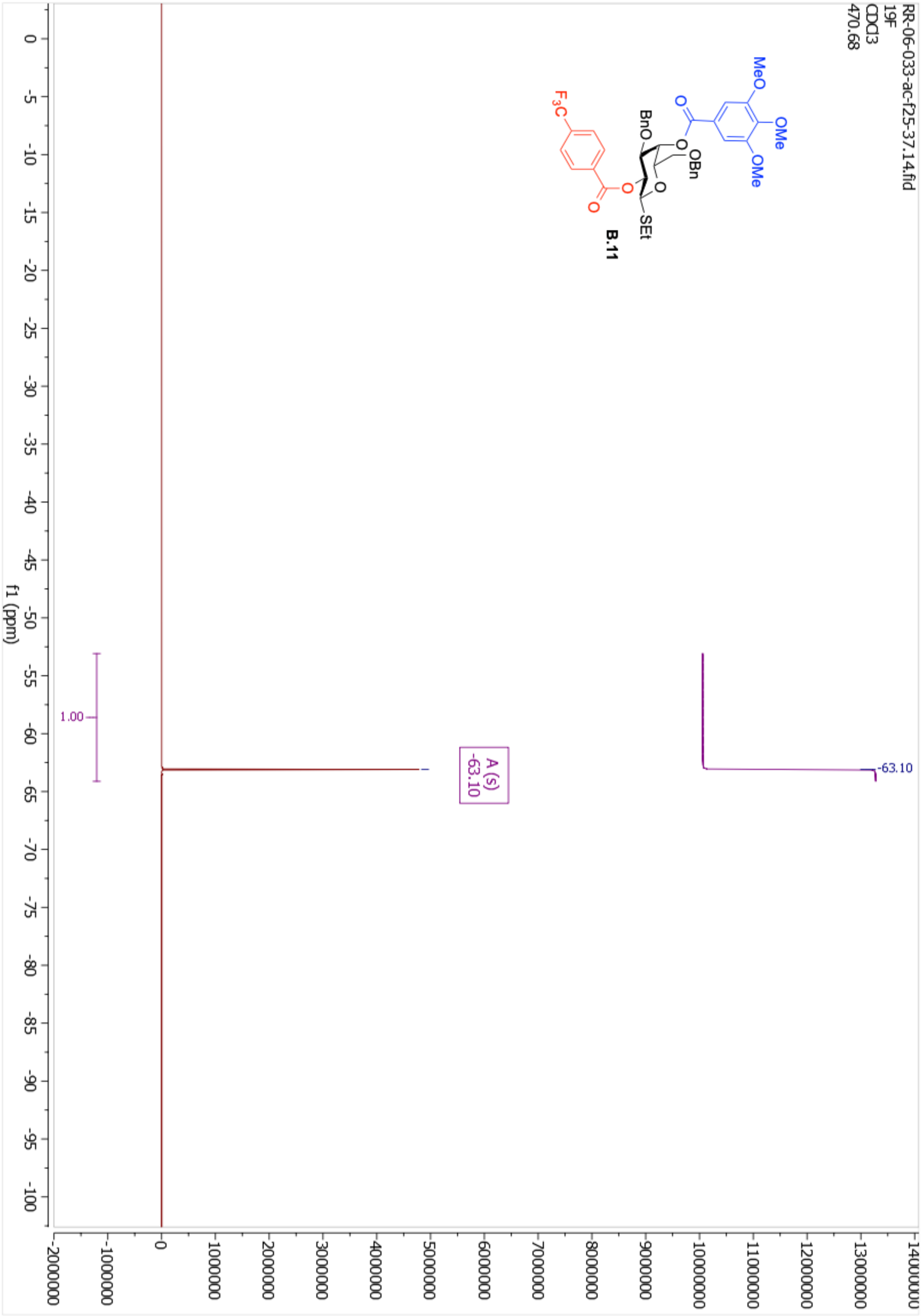
RR-06-033-ac-f25-37.14.fid
19F
CDCl3
470.68
B.11
-63.10
A (s)
-63.10
1.00
f1 (ppm)
